# Alkynes Electrooxidation to α,α-Dichloroketones in Seawater with Natural Chlorine Participation via Competitive Reaction Inhibition and Tip-Enhanced Reagent Concentration

**DOI:** 10.1021/acscentsci.3c01277

**Published:** 2023-12-22

**Authors:** Junwei Yao, Rong Yang, Cuibo Liu, Bo-Hang Zhao, Bin Zhang, Yongmeng Wu

**Affiliations:** Department of Chemistry, School of Science, Tianjin University, Tianjin 300072, China

## Abstract

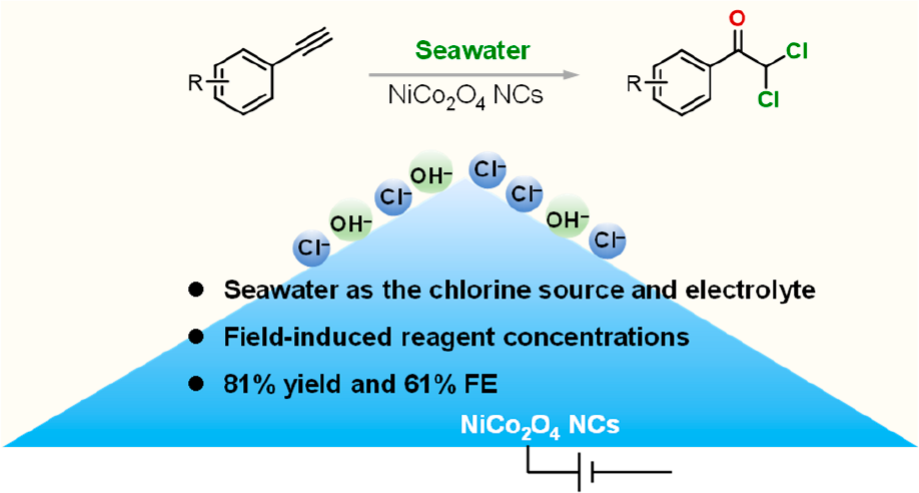

The traditional synthesis of α,α-dichloroketones usually requires corrosive chlorine, harsh reaction conditions, or excessive electrolytes. Here, we report an electrooxidation strategy of ethynylbenzenes to α,α-dichloroketones by directly utilizing seawater as the chlorine source and electrolyte solution without an additional supporting electrolyte. High-curvature NiCo_2_O_4_ nanocones are designed to inhibit competitive O_2_ and Cl_2_ evolution reactions and concentrate Cl^–^ and OH^–^ ions, accelerating α,α-dichloroketone electrosynthesis. NiCo_2_O_4_ nanocones produce 81% yield, 61% Faradaic efficiency, and 44.2 mmol g^_cat._–1^ h^–1^ yield rate of α,α-dichloroketones, outperforming NiCo_2_O_4_ nanosheets. A Cl^•^ radical triggered Cl^•^ and OH^•^ radical addition mechanism is revealed by a variety of radical-trapping and control experiments. The feasibility of a solar-powered electrosynthesis system, methodological universality, and extended synthesis of α,α-dichloroketone–drug blocks confirm its practical potential. This work may provide a sustainable solution to the electrocatalytic synthesis of α,α-dichloroketones via the utilization of seawater resources.

## Abstract 2

An *α*,*α*-dichloroketone electrosynthesis strategy by directly utilizing seawater as the chlorine source and electrolyte solution without an additional supporting electrolyte is reported.

## Introduction

α,α-Dichloroketones are essential structural motifs in various pharmaceutical chemicals and natural products.^1,2^ Traditional methods to synthesize α,α-dichloroketones mainly include the dichlorination of ketones and the oxydichlorination of alkynes (Supplementary Figure 1a,b).^3−5^ In these methods, excessive and corrosive chlorine (Cl_2_) or expensive/toxic organochlorination reagents, such as *N*-chlorosuccinimide (NCS) and trichloroisocyanuric acid (TCCA), are used as chlorine sources. Additionally, excess strong oxidants and high reaction temperatures are usually needed, causing concerns about safety, cost, and sustainability. Therefore, it is highly desirable to develop an alternative strategy to achieve a sustainable, mild, and efficient synthesis of α,α-dichloroketones.

Renewable electricity-driven transformation has emerged as an attractive strategy in synthetic chemistry.^6−16^ An advance has been made in using trichloromethane (CHCl_3_) as the chlorine source to realize the electrosynthesis of α,α-dichloroketones from alkynes in organic electrolyte solution (Supplementary Figure 1c).^17^ In this process, tetrabutylammonium iodide (TBAI) was added to serve as a nucleophile; thus, Cl^–^ was generated from CHCl_3_ by nucleophilic substitution with I^–^. Then, Cl^–^ was oxidized to chlorine radical (Cl^•^) at the anode, triggering the oxydichlorination reaction. However, the reaction rate was severely restricted by the slow Cl^–^ generation process. Additionally, carcinogenic CHCl_3_, toxic TBAI, and massive and expensive organic electrolytes are needed in the electrosynthesis process, causing sustainability and cost issues. Thus, it is highly significant to develop a sustainable electrolysis system for efficient α,α-dichloroketone electrocatalytic synthesis.

In the chlor-alkali process, using saturated NaCl as the electrolyte, Cl^•^ radicals are first produced by Cl^–^ ion electrooxidation and then self-coupled to form Cl_2_.^18,19^ Inspired by this process, we speculate that the Cl^•^ radicals generated in situ from Cl^–^ ion oxidation can trigger the oxydichlorination reaction for α,α-dichloroketone electrosynthesis. Additionally, seawater accounts for 96.5% of the Earth’s total water and is an almost inexhaustible resource.^20−22^ The dominant ions in seawater are Na^+^ and Cl^–^, accounting for approximately 3.5 wt %. This encourages us to consider utilizing Na^+^ and Cl^–^ as the conducting ions, Cl^–^ as the chlorine source, and H_2_O as the oxygen source; thus, the sustainable electrocatalytic synthesis of α,α-dichloroketones could be realized by directly using seawater as the electrolyte solution without an additional chlorine source and a supporting electrolyte.

**Figure 1:**
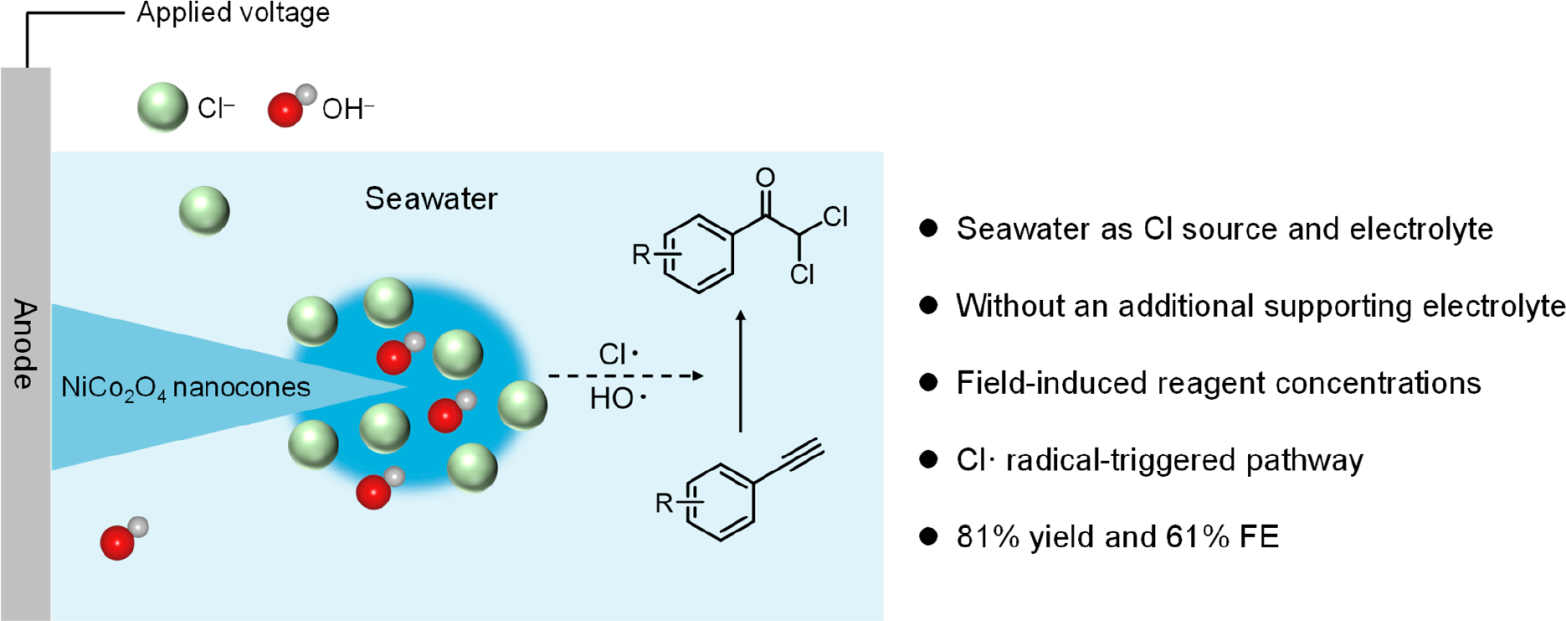
Schematic diagram of α,α-dichloroketone synthesis from seawater under ambient conditions.

Here, a strategy for the electrocatalytic synthesis of α,α-dichloroketones without an additional chlorine source and a supporting electrolyte is demonstrated (Figure 1). A high-curvature NiCo_2_O_4_ nanocone (NC) anode is proposed as a promising candidate to electrosynthesize α,α-dichloroketones from alkynes in seawater by analyzing the reaction process and possible competitive reactions. An 81% yield, 61% Faradaic efficiency (FE), and 44.2 mmol g^_cat._–1^ h^–1^ yield rate of α,α-dichloroketone electrocatalytic synthesis are achieved over the NiCo_2_O_4_ nanocones (NCs), outperforming NiCo_2_O_4_ nanosheets (NSs). A series of radical-trapping and control experiments confirm the Cl^•^ radical triggered mechanism. The solar energy powered electrosynthesis system on a flow reactor demonstrates the potential of this strategy. Taking the electrosynthetic product as the building block, an adrenocortical carcinoma treatment drug, mitotane, was synthesized. This sustainable system is suitable for synthesizing other α,α-dichloroketones with high yields.

## Results and Discussion

### Calculation-Assisted Electrocatalyst Screening and Design

**Figure 2:**
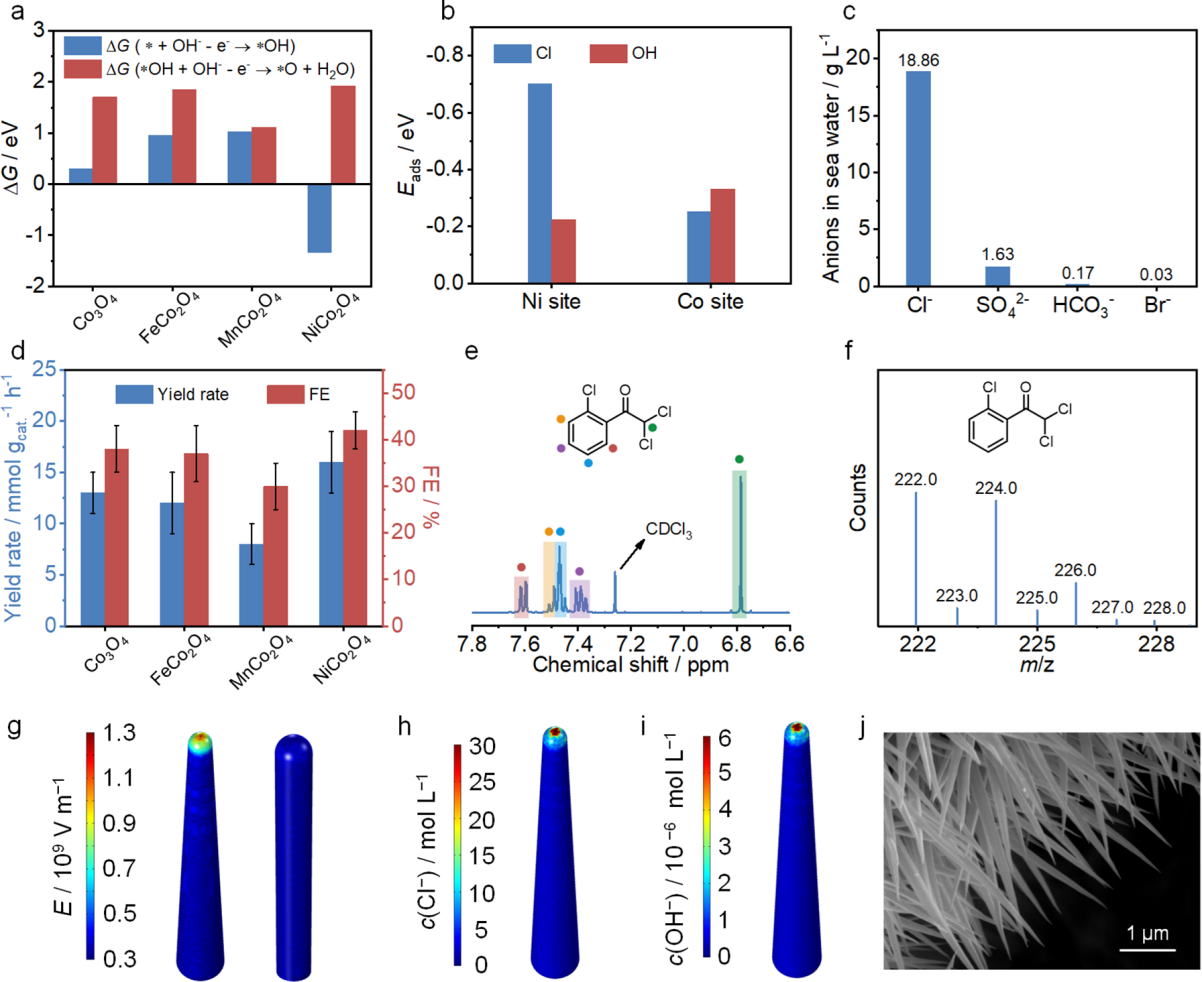
Electrocatalyst screen, design, and characterization. (a) Δ*G*(* + OH^–^ – e^–^ → *OH) and Δ*G*(*OH + OH^–^ – e^–^ → *O + H_2_O) over different anode materials. (b) Comparison of *E*_ads_ values for OH and Cl on Ni and Co sites. (c) Concentrations of the main anions in the natural seawater collected from the Bohai Sea, Tianjin, China. (d) Comparison of electrosynthesis performances at 1.30 V. (e) ^1^H NMR and (f) GC–MS detection of the products. (g) Electric field on the surface of NiCo_2_O_4_ samples with different tip radii of 5 (top) and 12 nm (bottom). (h) Surface Cl^–^ and (i) OH^–^ concentration distributions adjacent to the surface of NiCo_2_O_4_ NCs. (j) SEM image of NiCo_2_O_4_ NCs. Error bars correspond to the standard deviation (SD) of three independent measurements.

The first consideration in selecting a catalyst is its stability under electrooxidation conditions in the presence of seawater. It was learned from the electrolytic seawater oxidation reaction that cobalt-based oxides with spinel structures are good electrocatalytic oxidation catalysts with high stability^23−27^ and thus are considered candidates for alkyne oxydichlorination to synthesize α,α-dichloroketones. For a high reaction rate and FE α,α-dichloroketone electrosynthesis, three fundamental factors should be considered for a suitable electrocatalyst: (1) low activation energy for *OH formation (* + OH^–^ – e^–^ → *OH) but high Δ*G*(*OH + OH^–^ – e^–^ → *O + H_2_O) to prohibit the O_2_ evolution reaction (OER),^28−31^ (2) discontiguous Cl^–^ adsorption sites to suppress Cl_2_ evolution, and (3) concentrated reactant ions near the catalyst surface to accelerate mass transfer. First, four cobalt-based oxides with spinel structures (Co_3_O_4_, FeCo_2_O_4_, MnCo_2_O_4_, and NiCo_2_O_4_) that are commonly used in electrooxidation reactions are screened by density functional theory (DFT) calculations.^32,33^ Among these catalysts, NiCo_2_O_4_ has the lowest Δ*G*(* + OH^–^ – e^–^ → *OH) and the highest Δ*G*(*OH + OH^–^ – e^–^ → *O + H_2_O) (Figure 2a), which is favorable for producing *OH to synthesize α,α-dichloroketones but inhibits the competitive OER. Moreover, the adsorption energy (*E*_ads_) of Cl on Ni is much higher than that on Co, while OH shows the opposite trend (Figure 2b and Supplementary Figures 2–4). Thus, Cl preferentially adsorbs on Ni sites, and OH tends to adsorb on Co sites. This nonadjacent adsorption site can suppress the self-coupling of Cl^•^ to form Cl_2_.^11^

To verify the above analysis, we synthesized four cobalt-based spinel oxides and tested their α,α-dichloroketone electrosynthesis performances (Supplementary Figures 5–8). Natural seawater collected in the Bohai Sea was used as the electrolyte solution with a Cl^–^ ion concentration of ∼18 g L^–1^ (determined by ion chromatography; Figure 2c). 1-Chloro-2-ethynylbenzene was selected as the model substrate. After electrolysis for 10 min at 1.30 V vs Ag/AgCl, NiCo_2_O_4_ NSs show the highest α,α-dichloroketone yield rate (16 mmol g^_cat._–1^ h^–1^) and FE (42%), corresponding to the theoretical prediction (Figure 2d). The target product of α,α-dichloroketone was confirmed by ^1^H nuclear magnetic resonance (^1^H NMR) and gas chromatography–mass spectrometry (GC–MS) (Figure 2e,f). These results indicate that the electrosynthesis of α,α-dichloroketone using seawater as the electrolyte is workable and that NiCo_2_O_4_ is a good anode candidate.

High-curvature nanostructures can enhance the local electric field to enrich reactant ions near the electrode surface, thus accelerating the reaction rate.^34,35^ In our proposed reaction process, Cl^–^ and OH^–^ ions are needed for α,α-dichloroketone electrosynthesis. Thus, it is supposed that enhancing the local electric field of the anode can concentrate Cl^–^ and OH^–^ ions from the seawater electrolyte solution and thus promote α,α-dichloroketone electrosynthesis. This speculation was confirmed by the finite element method simulation. The electric field intensity was obviously enhanced as the radius decreased from 12 to 5 nm due to the migration of free electrons to the regions of the sharpest curvature on a charged metallic electrode (Figure 2g and Supplementary Figure 9).^36^ As a result, the concentration of Cl^–^ and OH^–^ ions in the Helmholtz layer of the electrical double layer increased by 60-fold at the nanocone tips compared with that of the bulk electrolyte solution (Figure 2h,i). Thus, a high-curvature nanostructure is expected to promote α,α-dichloroketone electrosynthesis.

### Electrocatalyst Synthesis and Characterizations

Self-supported NiCo_2_O_4_ NCs and NiCo_2_O_4_ NSs were successfully synthesized via a hydrothermal method followed by calcination in air.^37,38^ Scanning electron microscopy (SEM) and transmission electron microscopy (TEM) images show NiCo_2_O_4_ NCs and NiCo_2_O_4_ nanosheets (NSs) grown on the substrate uniformly with the nanocone and nanosheet morphologies (Figure 2j and Supplementary Figures 10 and 11). The lattice fringes with interplanar distances of 0.468 nm for NiCo_2_O_4_ NCs and 0.467 nm for NiCo_2_O_4_ NSs in the high-resolution TEM (HRTEM) images are both ascribed to the (111) plane of NiCo_2_O_4_ (Supplementary Figure 12).^37^ The energy dispersive X-ray (EDX) mapping images reveal uniformly dispersed Ni, Co, and O elements throughout the entire nanocones or nanosheets (Supplementary Figure 13). All the diffraction peaks in the X-ray diffraction (XRD) patterns of the NiCo_2_O_4_ NCs and NiCo_2_O_4_ NSs are indexed to the standard NiCo_2_O_4_ (JCPDS No. 20-0781) (Supplementary Figure 14).^37,38^ X-ray photoelectron spectroscopy (XPS) spectra for Ni 2p, Co 2p, and O 1s of the two materials show similar results. In Ni 2p, two spin–orbit doublets at 874.0 and 872.3 eV correspond to the characteristic Ni^2+^, while two shake-up satellites at 856.3 and 854.5 eV are ascribed to Ni^3+^ (Supplementary Figure 15).^37,38^ In Co 2p, the peaks at 797.1 and 795.0 eV are assigned to Co^2+^ and 781.7 and 779.9 eV correspond to Co^3+^ (Supplementary Figure 15).^37,38^ The peaks at 529.6 and 531.2 eV in O 1s are distributed to the metal–oxygen bonds and the oxygen defect sites (Supplementary Figure 15).^37,38^ These results indicate the successful synthesis of NiCo_2_O_4_ NCs and NSs.

### α,α-Dichloroketone Electrosynthesis Performance

**Figure 3:**
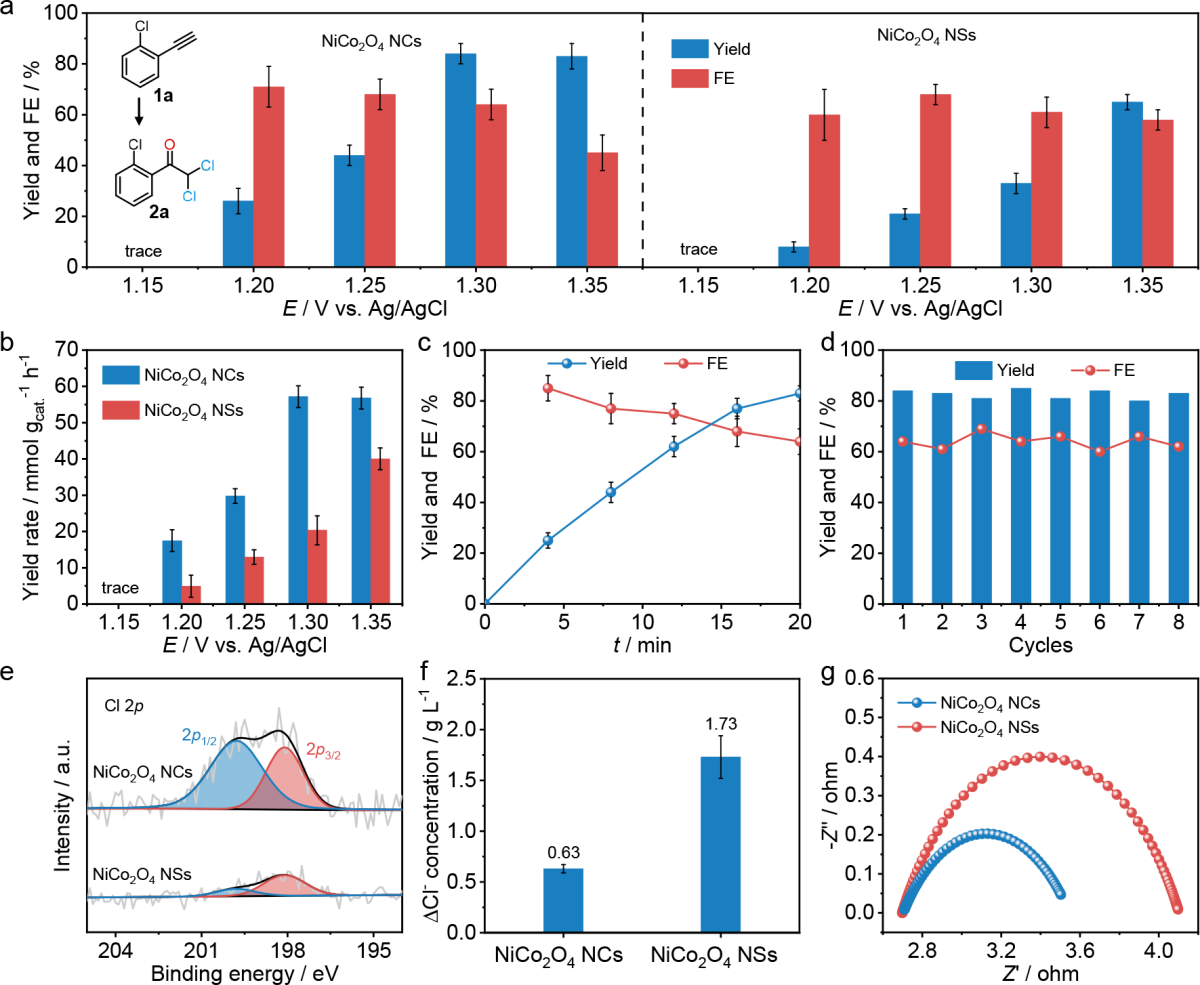
Eectrosynthesis performance. (a) Potential-dependent FEs and yields and (b) yield rates toward **2a**. (c) Time-dependent yields over NiCo_2_O_4_ NCs. (d) Cycle-dependent FEs and yields of **2a** electrosynthesis over NiCo_2_O_4_ NCs. (e) Cl 2p XPS spectra of NiCo_2_O_4_ NCs and NSs. (f) Field-induced Cl^–^ loss in the electrolytes caused by NiCo_2_O_4_ NCs and NSs. (g) Nyquist plots at 1.30 V vs Ag/AgCl of NiCo_2_O_4_ NCs and NSs. Error bars correspond to the SD of three independent measurements.

To simplify the performance and mechanism studies, 0.5 M NaCl, a common alternative to seawater for electrocatalytic investigation, was used as the electrolyte solution to replace seawater in the following study.^20−22^ The α,α-dichloroketone electrosynthesis performance over NiCo_2_O_4_ NCs and NSs was tested in a divided three-electrode system. 1-Chloro-2-ethynylbenzene (**1a**) was chosen as the model substrate. Acetonitrile was added as a cosolvent to increase the solubility of the substrate. All potentials refer to Ag/AgCl unless otherwise noted. Linear sweep voltammetry (LSV) curves show a remarkable increase in current density for NiCo_2_O_4_ NCs compared with NSs (Supplementary Figure 16). Potential-dependent tests indicate that NiCo_2_O_4_ NCs present a superior performance compared to NiCo_2_O_4_ NSs in the potential range 1.15–1.35 V (Figure 3a and Supplementary Figure 17). NiCo_2_O_4_ NCs exhibit the optimum performance of 84% yield, 64% FE, and 57.2 mmol g^_cat._–1^ h^–1^ yield rate at a potential of 1.30 V. The yield rate of NiCo_2_O_4_ NCs is nearly 3 times that of NiCo_2_O_4_ NSs at 1.30 V, indicating the promoting effect of the high-curvature structure for this electrocatalytic reaction (Figure 3b). Time-dependent experiments show that 0.1 mmol of **1a** could be consumed totally, and an 84% **2a** yield was obtained within 20 min (Figure 3c), which is far faster than the I^–^-mediated electrosynthesis method in organic electrolyte solution (0.3 mmol of substrate was consumed within 6 h).^17^ Furthermore, no performance degeneration is observed during eight cycles of electrocatalytic tests, and the morphology and composition can be maintained as before, suggesting that NiCo_2_O_4_ NCs are a promising candidate for α,α-dichloroketone electrosynthesis with high intrinsic activity and durability (Figure 3d and Supplementary Figures 18–20). In addition, the performances of NiCo_2_O_4_ NCs and NiCo_2_O_4_ NSs were tested in seawater. The performance of NiCo_2_O_4_ NCs reaches 81% yield, 61% FE, and 44.2 mmol g^_cat._–1^ h^–1^ yield rate, which far exceeds that of NiCo_2_O_4_ NSs (Supplementary Figure 21). This further confirms the promotional effect of the high-curvature structure for this electrocatalytic reaction.

Further experiments were conducted to understand the promotion origin of the high-curvature structure in α,α-dichloroketone electrosynthesis. First, the electrochemical surface areas (ECSAs) of NiCo_2_O_4_ NCs and NSs were evaluated by double layer capacitance (*C*_dl_) measurements. The *C*_dl_ of NiCo_2_O_4_ NCs is 18.05 mF cm^–2^, slightly higher than that of NiCo_2_O_4_ NSs (16.52 mF cm^–2^) (Supplementary Figure 22). However, the difference in the ECSA (ECSA_NCs_:ECSA_NSs_ = 1.1) of the two catalysts is much smaller than that of the yield rate (*Y*, *Y*_NCs_:*Y*_NSs_ = 3.1), indicating that the performance improvement is mainly from the intrinsic activity of the catalyst rather than the ECSA increase. Furthermore, it is supposed that the enhanced positive electric field around the tips of NiCo_2_O_4_ NCs can concentrate electronegative OH^–^ and Cl^–^ ions. It was verified by experiments. A constant voltage of 1.30 V was applied to the NiCo_2_O_4_ NCs and NiCo_2_O_4_ NSs for 20 min, and then the two electrodes were washed with deionized water and dried under vacuum for characterization. XPS measurements (Figure 3e) show that the NiCo_2_O_4_ NCs exhibit two typical Cl 2p XPS peaks at 198.1 eV (Cl 2p_3/2_) and 199.8 eV (Cl 2p_1/2_),^9^ while the NiCo_2_O_4_ NSs exhibit much weaker Cl 2p peaks at the same position. Meanwhile, the adsorption of Cl^–^ ions was quantified by measuring the Cl^–^ ion concentration variation of the electrolyte solution. A larger Cl^–^ ion concentration difference value was observed before and after electrolysis when NiCo_2_O_4_ NCs was used as the electrode, demonstrating that NiCo_2_O_4_ NCs adsorb more Cl^–^ ions than NiCo_2_O_4_ NSs (Figure 3f). Moreover, after electrolysis for 20 min at 1.30 V, the electrolyte solution pH values decreased to 1.73 and 1.97 for NiCo_2_O_4_ NCs and NiCo_2_O_4_ NSs, respectively. The lower pH indicates that more OH^–^ ions are consumed, caused by the ion enrichment effect of NiCo_2_O_4_ NCs. These results experimentally prove that the high-curvature nanostructure can concentrate Cl^–^ and OH^–^ ions, as predicted by theoretical calculations. Additionally, NiCo_2_O_4_ NCs show a much smaller charge transfer resistance (*R*_ct_) in electrochemical impedance spectra, reflecting an acceleration of the charge transfer process at electrode/solution interfaces over NiCo_2_O_4_ NCs (Figure 3g). Therefore, the concentrated reactant ions and accelerated charge transfer around the tips of NiCo_2_O_4_ NCs conjointly promote the α,α-dichloroketone electrosynthesis performance.

### Mechanistic Study

Three possible pathways are considered to trigger the oxydichlorination of alkynes (Supplementary Figure 23): (1) The alkynyls on alkynes are first electrooxidized to form carbocations and then react with Cl^–^ to form vinyl radicals. (2) Cl^–^ ions are first oxidized to Cl_2_ and dissolved in water to form ClO^–^, which acts as an oxidation and chlorination agent to react with alkynes. (3) Cl^–^ ions are initially electrooxidized to Cl^•^ radicals, which attack the α-carbon of alkynes to form vinyl radicals for the following transformation.

**Figure 4:**
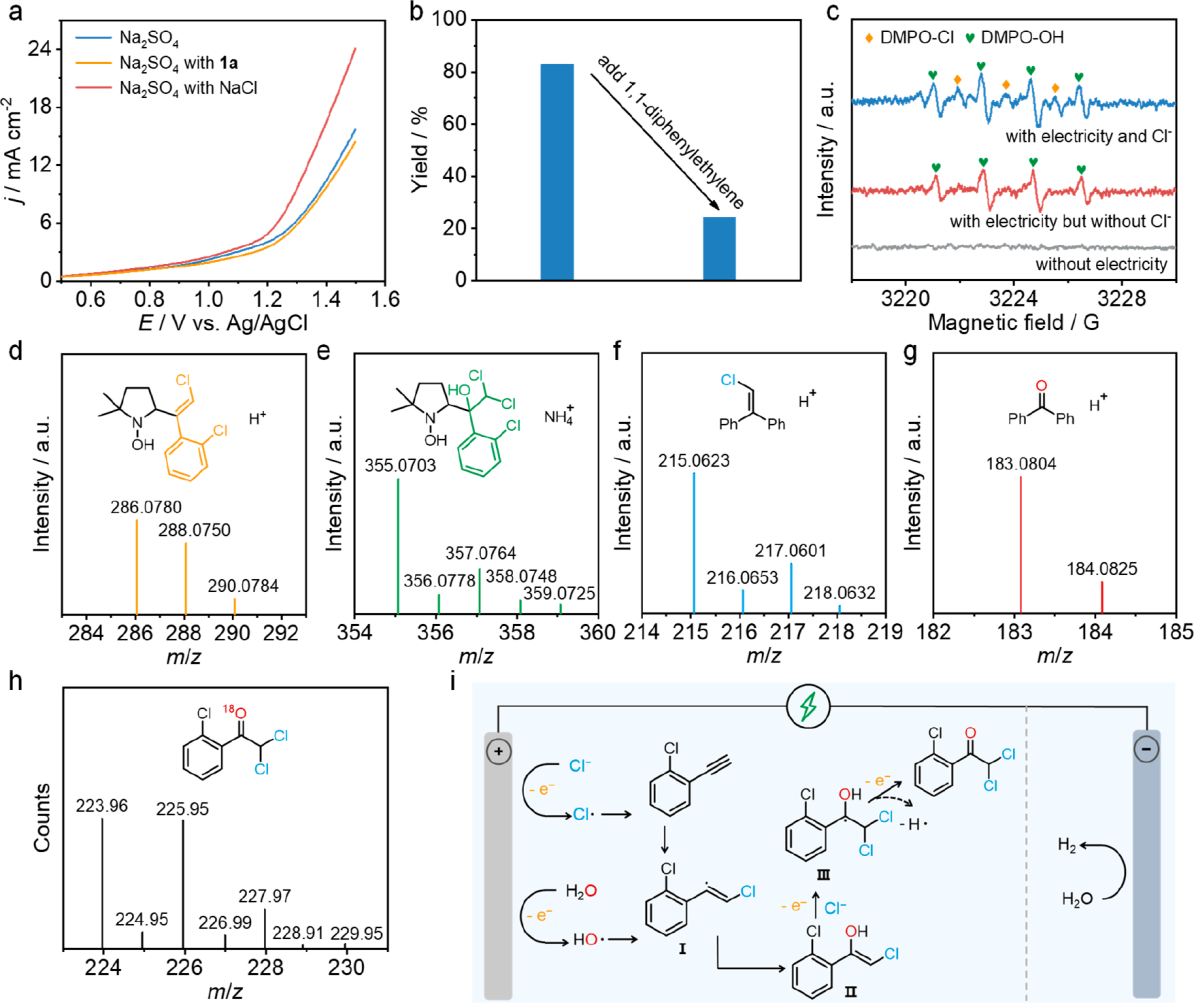
Mechanistic studies. (a) LSV curves measured in 0.5 M Na_2_SO_4_ solution. (b) Comparison of **2a** yields with and without 1,1-diphenylethylene in 0.5 M NaCl solution. (c) EPR trapping for radicals during the electrosynthesis process over NiCo_2_O_4_ NCs. HR–MS analysis of the spin-trapping experiment of (d, e) carbon radicals, (f) chlorine radicals, (g) hydroxyl radicals, and (h) ^18^O-labeled product. (i) A proposed possible reaction mechanism.

Control experiments and radical-trapping experiments were performed to identify the pathway for α,α-dichloroketone electrosynthesis. First, LSV curves were tested in 0.5 M Na_2_SO_4_ (Figure 4a). A higher current density and lower initial potential were observed after introducing 0.1 mmol of NaCl. However, when 0.1 mmol of **1a** was added, no current density increase was observed. These results indicate that Cl^–^ ion electrooxidation occurs more easily than the OER, while **1a** electrooxidation may have difficulty taking place in the tested potential range. This was further confirmed by the electrolysis experiment at 1.30 V in 0.5 M Na_2_SO_4_ containing 0.1 mmol of **1a.** No **1a** oxidative product was detected after the reaction (Supplementary Figure 24a). Moreover, a radical scavenger of 1,1-diphenylethylene was added during the reaction, and the hydroxyl radical (OH^•^) was trapped, while no carbon radical (C^•^) was captured (Supplementary Figure 24b). These results prove that H_2_O can be oxidized to OH^•^, but **1a** cannot be electrooxidized under the tested potential. Thus, path 1 can be excluded.

To see whether the reaction occurs through path 2, **1a** was added to 0.5 M NaCl containing 0.5 M ClO^–^, and no oxydichlorination product was probed without bias (Supplementary Figure 25). Therefore, path 2 is not the possible path.

Further experiments were performed to explore whether α,α-dichloroketone electrosynthesis occurs via path 3. When 1,1-diphenylethylene was added as a radical scavenger during the reaction process, the yield of **2a** decreased by 59% with Cl^•^ and OH^•^ radicals trapped, indicating the radical-mediated pathway for **2a** electrosynthesis (Figure 4b and Supplementary Figure 26). This result is further confirmed by electron paramagnetic resonance (EPR) measurements (Figure 4c). The characteristic EPR signals of the 5,5-dimethyl-1-pyrroline *N*-oxide (DMPO)–Cl^•^ (marked by diamond symbols) and DMPO–OH^•^ adducts (marked by heart symbols) were detected during the α,α-dichloroketone electrosynthesis process when 0.5 M NaCl was used as the electrolyte solution. However, C^•^ radicals failed to be detected in EPR measurements, probably due to the mutual interference of the different radical signals and the short life span of C^•^ radicals. Thus, we combined DMPO trapping experiments with high-resolution mass spectrometry (HR–MS) to identify the C^•^ radical related intermediates. The vinyl radical and ketyl radicals were captured by DMPO, and the *m*/*z* values of the compounds were determined by HR–MS to be 286.0780 and 355.0703, respectively (Figure 4d,e). Cl^•^ and OH^•^ were captured by 1,1-diphenylethylene, giving corresponding *m*/*z* values of 215.0623 and 183.0804, respectively (Figure 4f,g). These results prove that the electrosynthesis undergoes a radical mechanism triggered by Cl^•^, as proposed in path 3. Furthermore, the oxygen source for **2a** electrosynthesis is from H_2_O, as confirmed by the ^18^O isotope labeling experiment (Figure 4h).

On the basis of the above discussion, a possible reaction mechanism is proposed (Figure 4i). Cl^•^ radicals are initially produced by the electrooxidation of Cl^–^ ions at the anode, which trigger the oxydichlorination reaction by attacking the alkynyl of **1a** to form vinyl radical **I**. The resulting radical intermediate **I** was then subjected to nucleophilic attack by OH^•^, generated from H_2_O splitting, to produce enol **II**. The reason for intermediate **I** integrating with OH^•^ rather than the more concentrated Cl^•^ is that OH^•^ has a lower binding energy to **I**, as suggested in the theoretical calculation (Supplementary Figure 27 and Supplementary Note 1). Finally, enol **III** rapidly combines with the chlorine radical and is then further oxidized, affording the final oxydichlorination product **2a**, as well as a proton.

### Utility and Universality Studies

**Figure 5:**
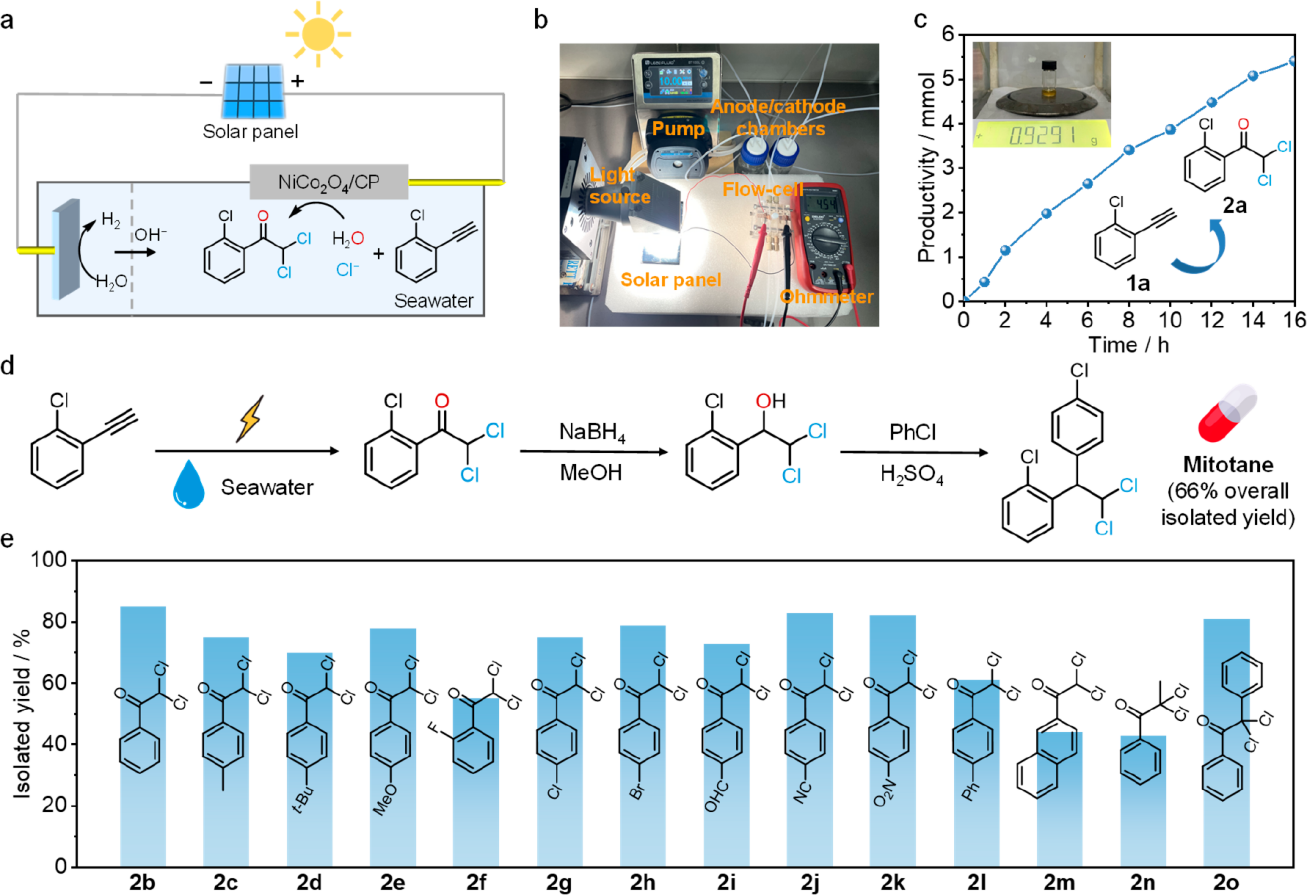
Universality study. (a) Schematic illustration of the solar-powered electrosynthesis system. (b) Photograph of the solar-driven electrosynthesis system under AM 4.5G (∼300 mW cm^–2^) illumination. (c) Productivity of the products in the solar-driven electrosynthesis system. (d) Mitotane synthesis using α,α-dichloroketone as the building block. (e) Synthesis of other α,α-dichloroketones through our electrosynthesis strategy. Isolated yields of α,α-dichloroketones are reported.

A solar-powered electrosynthesis system was designed and assembled (Figure 5a,b). A solar panel was used to provide constant bias, a homemade flow cell with NiCo_2_O_4_ NCs was employed as the anode, and seawater was directly utilized as the electrolyte and chlorine and oxygen source. The solar-powered electrosynthesis system successfully achieved the gram-scale electrosynthesis of α,α-dichloroketone under AM 4.5G illumination (300 mW cm^–2^) (Figure 5c). This demonstrates the potential of the electrosynthesis strategy for the sustainable production of organochlorides from seawater. Moreover, taking the electrosynthetic product as the building block, mitotane, the only FDA-approved drug for adrenocortical carcinoma treatment, was successfully synthesized with a 66% overall isolated yield (Figure 5d). This highlights the application potential of our electrosynthesis method in drug synthesis. Furthermore, the universality of this electrosynthesis strategy for α,α-dichloroketones was examined (Figure 5e). A series of alkynes bearing electron-donating and electron-withdrawing groups on the phenyl ring were all efficiently transformed to the corresponding α,α-dichloroketone products. Moreover, internal alkynes were also applicable for the oxydichlorination with good yield. These results highlight the applicability of electrosynthesis system without additional chlorine sources and electrolytes for electrocatalytic oxydichlorination.

## Conclusions

In summary, we demonstrate the efficient electrosynthesis of α,α-dichloroketones by directly using seawater as the chlorine source and electrolyte solution. A NiCo_2_O_4_ NC anode was designed by analyzing the reaction process, which can inhibit the competitive O_2_ and Cl_2_ evolution reactions and concentrate Cl^–^ and OH^–^ ions, accelerating α,α-dichloroketone electrosynthesis. NiCo_2_O_4_ NCs yield α,α-dichloroketone with 81% yield, 61% FE, and 44.2 mmol g^_cat._–1^ h^–1^ yield rate at the optimum potential of 1.30 V, which is superior to the performance of NiCo_2_O_4_ NSs. A mechanistic study revealed that this reaction is triggered by the attack of Cl^•^ radicals on alkynes, followed by OH^•^ and another Cl^•^ radical addition. The key Cl^•^, OH^•^, and carbon radical species were identified by EPR and HR–MS. Additionally, a solar-powered electrosynthesis system was designed and achieved gram-scale α,α-dichloroketone electrosynthesis. Moreover, an adrenocortical carcinoma treatment drug, mitotane, was synthesized with the use of the obtained α,α-dichloroketones as building blocks. Furthermore, 14 other examples of functionalized alkynes were oxydichlorinated in our electrosynthesis system, highlighting the universality of our strategy. This work not only opens up a sustainable strategy to synthesize organochlorides but also provides a new avenue for the direct utilization of abundant seawater.

## Methods

### Synthesis of NiCo_2_O_4_Nanocones (NCs) and NiCo_2_O_4_Nanosheets (NSs)^37,38^

In situ growth of NiCo_2_O_4_ nanocones on as-treated carbon paper (CP) was carried out by a general hydrothermal method followed by subsequent calcination. Specifically, 2 mmol of Co(NO_3_)_2_·6H_2_O along with 1 mmol of Ni(NO_3_)_2_·6H_2_O were dissolved in 40 mL of DI water, followed by the addition of 3 mmol of NH_4_F and 6 mmol of urea into the solution with continuous stirring. After transfer of the solution into a 50 mL Teflon-lined autoclave and immersion of a piece of as-treated CP (2 × 3 cm^2^) into the solution, the autoclave was properly sealed and treated at 100 °C for 12 h. After cooling to room temperature, the sample was washed with DI water and ethanol several times and then dried in a vacuum drying oven at 60 °C overnight. Finally, the dried sample was annealed in a tube furnace at 350 °C for 2 h under an air atmosphere. The loading mass of NiCo_2_O_4_ NCs was 4.4 mg cm^–2^. NiCo_2_O_4_ nanosheets were synthesized by a similar method, except 12 mmol of hexamethylene-tetramine was used to replace urea. The loading mass of NiCo_2_O_4_ NSs was 4.9 mg cm^–2^_._

### Electrochemical Measurements

Electrochemical tests were carried out using a CS350MA (CorrTest, Wuhan) electrochemical workstation in a two-chamber electrochemical cell consisting of a working electrode, a Pt plate counter electrode, and a Ag/AgCl reference electrode. All the potentials in this work were referenced to Ag/AgCl without *iR* correction unless otherwise stated. The cathode cell (8 mL) and anode cell (8 mL) contained seawater/0.5 M NaCl (8.0 mL) and a mixed solution of 0.5 M NaCl (6.0 mL) and acetonitrile (2.0 mL) with 0.1 mmol of **1a** was dissolved. Then, chronoamperometry or chronopotentiometry was carried out under magnetic stirring (800 rpm). After the reactions were finished, dodecane was added to the reaction system as an internal standard. Then, the solution at the anode cell was extracted with dichloromethane (DCM) and tested using GC to calculate the product yield. The double layer capacitance (*C*_dl_) was calculated from the slope of the linear fit of plots of current density versus scan rate. The cyclic voltammograms (CVs) were recorded in the non-Faradaic region, where charging of the double layer is responsible for the current density.

## Supporting Information Available

Additional characterizations, performance measurements, theoretical models, and ^1^H and ^13^C NMR characteristic spectra (PDF)
