# Supplementary material for: Alkynes Electrooxidation to α,α-Dichloroketones in Seawater with Natural Chlorine Participation via Competitive Reaction Inhibition and Tip-Enhanced Reagent Concentration
Source: ACS Cent Sci. 2023 Dec 22;10(1):155–62. doi: 10.1021/acscentsci.3c01277 (PMC10823507; doi:10.1021/acscentsci.3c01277)
Supplement: Supplementary file 1 — oc3c01277_si_001.pdf [file oc3c01277_si_001.pdf]

Supplementary Information

Alkyne Electrooxidation to *α,α*-Dichloroketones in Seawater with Natural Chlorine Participation via Competitive Reaction Inhibition and Tip-Enhanced Reagent Concentration

Junwei Yao[1,2], Rong Yang[1,2], Cuibo Liu[1], Bo-Hang Zhao[1], Bin Zhang[1,*], and Yongmeng Wu[1,*]

[1]Department of Chemistry, School of Science, Tianjin University, Tianjin 300072, China

[2]These authors contributed equally: Junwei Yao, Rong Yang

*E-mail: ymwu01@tju.edu.cn (Y.W.); bzhang@tju.edu.cn (B.Z.)

Synthesis of Co_3O_4 NSs[1]

$Co(NO_3)_2 \cdot 6H_2O$ (2 mmol) was dissolved in 40 mL of DIW, followed by the addition of urea (10 mmol) under stirring. The mixture was stirred for 30 min and then transferred into a 50 mL Teflon-lined stainless autoclave. The fresh-treated carbon paper (2 ×3 cm) was immersed into the autoclave and then sealed and heated at 120 ℃ for 6 h. After cooling to room temperature, the sample was washed with DIW and ethanol several times and dried at 60 ℃ for 24 h. Finally, the dried sample was annealed at 350 ℃ for 2 h. The loading mass of Co_3O_4 NSs was 3.5 mg cm^{-2}.

Synthesis of $FeCo_2O_4$ NSs[2]

$Fe(NO_3)_2 \cdot 9H_2O$ (1 mmol) and $Co(NO_3)_2 \cdot 6H_2O$ (2 mmol) were dissolved in 40 mL of DIW, followed by the addition of urea (10 mmol) under stirring. The mixture was stirred for 30 min and then transferred into a 50 mL Teflon-lined stainless autoclave. The fresh-treated CP (2 ×3 cm^2) was immersed into the autoclave and then sealed and heated at 140 ℃ for 6 h. After cooling to room temperature, the sample was washed with DI water and ethanol several times and dried at 60 ℃ for 24 h. Finally, the dried sample was annealed at 350 ℃ for 2 h. The loading mass of $FeCo_2O_4$ NSs was 5.3 mg cm^{-2}.

Synthesis of $MnCo_2O_4$ NSs[1]

$Mn(NO_3)_2 \cdot 6H_2O$ (1 mmol) and $Co(NO_3)_2 \cdot 6H_2O$ (2 mmol) were dissolved in 40 mL of DIW, followed by the addition of urea (10 mmol) under stirring. The mixture was stirred for 30 min and then transferred into a 50 mL Teflon-lined stainless autoclave. The fresh-treated CP (2 ×3 cm^2) was immersed into the autoclave and then sealed and heated at 120 ℃ for 6 h. After cooling to room temperature, the sample was washed with DIW and ethanol several times and dried at 60 ℃ for 24 h. Finally, the dried sample was annealed at 350 ℃ for 2 h. The loading mass of $MnCo_2O_4$ NSs was 5.4 mg cm^{-2}.

Characterizations

Scanning electron microscopy (SEM) images were taken by an FEI Apreo S LoVac microscope. Transmission electron microscopy (TEM) images were taken by an FEI Tecnai G2 F20 microscope. The X-ray diffraction (XRD) patterns were recorded in the range of 10 to 90° using a Rigaku Smartlab9KW diffraction system with a Cu *K*α source (λ = 1.54056 Å). The X-ray photoelectron spectra (XPS) were obtained on a Thermo Fisher ESCALAB-250Xi photoelectron spectrometer using a monochromatic Al Kα X-ray beam (1486.60 eV). All the peaks were calibrated by the binding energy of 284.8 eV of the C 1*s* spectrum. The NMR spectra were recorded on a JEOL JNM-ECZ400S/L1 instrument at 400 MHz (^{1}H-NMR) and 101 MHz (^{13}C-NMR) with $CDCl_3$ as the solvent. Chemical shifts were reported in parts per million (ppm) downfield from internal tetramethylsilane. Multiplicity was indicated as follows: *s* (singlet), *d* (doublet), *t* (triplet), *m* (multiplet), *br* (broad). Coupling constants were reported in hertz (Hz). Gas chromatography−mass spectrometry (GC−MS) was carried

out with TRACE DSQ. The gas chromatograph (GC) was measured on an Agilent 7890A with thermal conductivity (TCD), flame ionization detector (FID) and HP-5 capillary column (0.25 mm in diameter, 30 m in length). The injection temperature was set at 300 ℃. Identification of the reactants and products was performed by gas chromatography−mass spectrometry (Agilent, 8860GC-5977MS) with an HP-5MS capillary column (0.25 mm in diameter, 30 m in length). The injection temperature was set at 300 ℃. Nitrogen was used as the carrier gas at 1.5 mL min^{-1}. Accurate mass measurements of the products were obtained via high−resolution mass spectrometry (HR−MS, ESI) on an Agilent 6550 QTOF. Radicals were investigated with electron spin resonance (EPR) spectroscopy (JES-FA200, JEOL, Japan).

Surface-adsorbed Cl^- ions

$NiCo_2O_4$ NCs and $NiCo_2O_4$ NSs were run in the initial NaCl-containing solution at 1.30 V vs. Ag/AgCl. After 20 min, the Cl^- ion concentrations in the electrolytes were detected by ion chromatography (IC), and the Cl^- ions adsorbed on the catalyst were detected by XPS Cl 2*p*. The adsorbed Cl^- was then estimated based on the loss of Cl^- concentrations in the electrolytes and the peak area of Cl 2*p* in XPS.

Quasi-in situ EPR trapping of the radicals

Chlorine radicals and hydroxide radicals are trapped by the addition of 5,5-dimethyl-1-pyrroline-N-oxide (DMPO) in a solution of 0.5 M NaCl. A $NiCo_2O_4$ NCs working electrode and a Ag/AgCl reference electrode were put into the anodic chamber, and a Pt plate counter electrode was inserted into the cathodic chamber. Chronoamperometry was carried out at a given constant potential of 1.30 V vs. Ag/AgCl for 10 min, and then 0.1 mM DMPO was added and stirred for 1 min. After that, the solution was quickly removed for the EPR test. The hydroxide radicals are trapped by DMPO in the same method, except that the solution is converted to 0.5 M Na_2SO_4.

Product identification and quantification.

The products in the electrolyte were identified by NMR spectroscopy and GC−MS. Product yield was quantified by GC with dodecane as the internal standard. The amount of the analyte was calculated based on the area ratio of the analyte peak to that of the internal standard. For the identification and quantification of organic products, after electrolysis, after the reactions finished, the products were extracted by dichloromethane (DCM) and analyzed by NMR spectroscopy and GC−MS.

The yield was calculated by the following equation:

$$\text{Yield (\%)} = \frac{\text{mol of the formed product}}{\text{mol of the initial substrate}} \times 100\%$$

The yield rate was calculated by the following equation:

$$\text{Yield rate (mmol } g_{cat.}^{-1}\text{ h}^{-1}) = \frac{\text{mol of the formed product}}{\text{mol of the initial substrate} \times t \times \text{m}} \times 100\%$$

where t is the reaction time and m is the mass of catalyst over the electrode.

The Faradaic efficiency (FE) is the ratio of the number of electrons transferred for the formation of each product to the total amount of electricity passing through the circuit. The FE for the products was calculated using the following equation:

$$\text{FE (\%)} = \frac{b \times n \times F}{Q} \times 100\%$$

where F is the Faradaic constant, Q is the electric charge, n is the number of moles of generated products, and b is the electron transfer number.

In this paper, error bars correspond to the standard deviation of three independent measurements.

Synthesis of Mitotane[3]

2,2-Dichloro-1-(2-chlorophenyl)ethan-1-one **2a** (1.0 mmol) was dissolved in methanol (10 mL) and continuously stirred for 10 min at 0 ℃. Then, $NaBH_4$ (1.5 equiv.) was added to the mixture. After completion of the reaction, the mixture was quenched by 1 N aqueous HCl. The mixture was purified by column chromatography on silica gel to give a colorless oil in 85% yield. Subsequently, 0.3 mmol of product was dissolved in chlorobenzene (2 mL), and concentrated H_2SO_4 (1 mL) was added dropwise into the mixture and stirred at room temperature for 30 min. Then, the mixture was quenched with saturated aqueous $NaHCO_3$ and extracted with DCM. The organic layer was purified by column chromatography on silica gel to give Mitotane as colorless oil in 93% yield.

Finite element simulations

Nernst–Planck-Poisson (NPP) calculations[4,5] of the Gouy-Chapman model[6] were employed using COMSOL to elucidate the underlying mechanisms of charge transfer and storage, as well as ion diffusion, governed by the Poisson equation[7]:

$$\nabla \cdot (\varepsilon_0 \varepsilon_r \nabla \varphi) = -\sum_i z_i e C_i \quad (1)$$

and the Nernst-Plank equation[9]:

$$\frac{\partial C_i}{\partial t} = \nabla \cdot \left[D_i \nabla C_i + \frac{D_i C_i}{k_B T} z_i e \nabla \varphi \right] \quad (2)$$

where ε_0 is the permittivity of vacuum, ε_r is the relative permittivity of the medium, φ is the electrostatic potential, D_i is the diffusivity of chemical species i, C_i is the density of the species, z_i is the valency of the species, e is the elementary charge, k_B is Boltzmann's constant, and T is temperature.

The geometries arising from the experimental TEM were used in the finite element model (FEM) with a conical bottom diameter of 24 nm, top diameter of 10 nm, and height of 180 nm. In addition, a less pointed shape was constructed with a top diameter of 24 nm to compare the influence of the radius of curvature. All of the models were immersed in 0.5 M NaCl aqueous electrolyte and subjected to a voltage of 1.30 V. The diffusion coefficients D of the sodium, chloride, hydrogen, and hydroxyl ions

were taken as 1.33×10^{-9}, 2.03×10^{-9}, 7.10×10^{-9}, and 5.30×10^{-9} m^2 s^{-1}, respectively. The absolute temperature *T* was taken as 298.15 K. The system mesh was set as a free tetrahedral mesh.

DFT calculations

All DFT calculations were performed using the Vienna Ab initio Simulation Package (VASP)[8]. The projector augmented wave (PAW)[9] pseudopotential with the PBE[10] generalized gradient approximation (GGA) exchange correlation function was utilized in the computations. Ueff (U-J) values of 3.32, 6.4, 4.2 and 3.9 eV were applied for the Co, Ni, Fe and Mn 3d states, respectively[11,12]. The cutoff energy of the plane wave basis set was 500 eV, and a Monkhorst-Pack mesh of 3×3×1 was used in K-point. The long-range dispersion interaction was described by the DFT-D3 method. The electrolyte was incorporated implicitly with the Poisson-Boltzmann model implemented in VASPsol[13]. The relative permittivity of the media was chosen as ε_r = 78.4, corresponding to that of water. All structures were spin polarized, all atoms were fully relaxed with an energy convergence tolerance of 10^{-5} eV per atom, and the final force on each atom was < 0.05 eV $Å^{-1}$. The most common structure and crystal plane of the material are used in the calculation models. For example, spinel structures such as $FeCo_2O_4$ and $MnCo_2O_4$ use the (311) crystal plane. All periodic slabs have a vacuum layer of at least 15 Å. All atoms could relax during geometry optimization.

The adsorption energy of the reaction intermediates can be computed using Equation (1):

$$\Delta G_{ads}=(E_{*ads} - E_{*} - E_{ads}) + \Delta E_{ZPE} - T\Delta S \quad (1)$$

where ads = (*OH or *O), $(E_{*ads} - E_{*} - E_{ads})$ is the binding energy, ΔE_{ZPE} is the zero-point energy change, and ΔS is the entropy change. In this work, the values of ΔE_{ZPE} and ΔS were obtained by vibration frequency calculation.

The OER mechanism used in calculations (2) and (3):

$$* + OH^{-} \rightarrow *OH + e^{-} \quad (2)$$

$$*OH + OH^{-} \rightarrow *O + H_2O + e^{-} \quad (3)$$

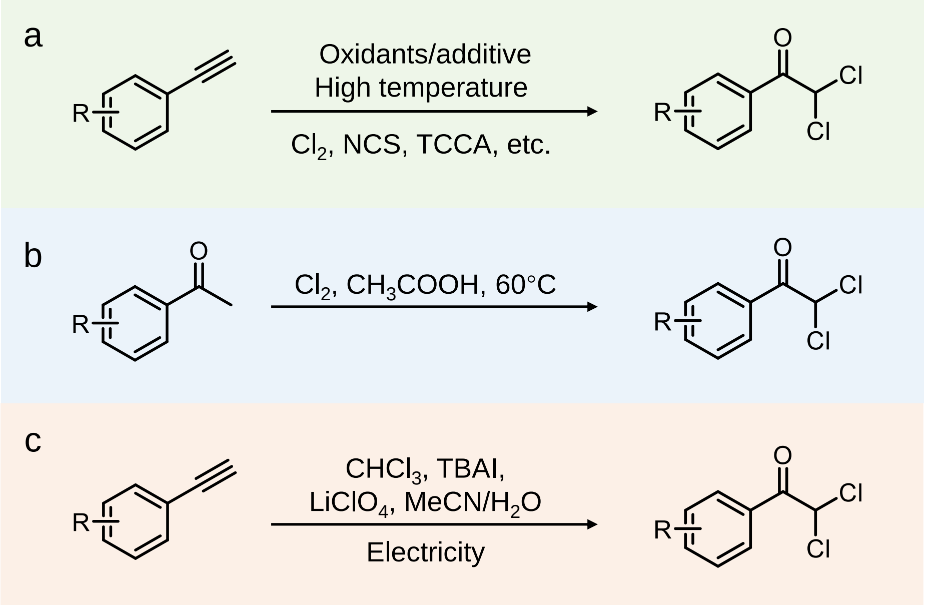

Supplementary Figure 1. a, Dichlorination of ketones method. **b**, Oxydichlorination of alkynes method. **c**, Reported electrochemical method.

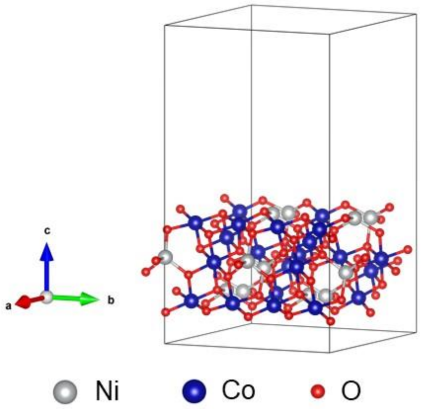

Supplementary Figure 2. The calculation model $NiCo_2O_4$.

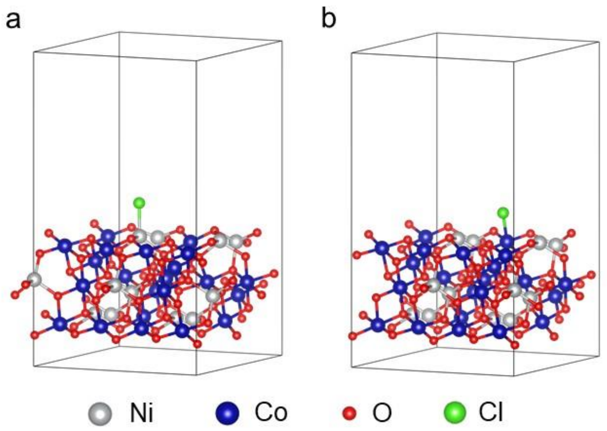

Supplementary Figure 3. The calculation model of Cl absorbed on **a**, Ni site and **b**, Co site.

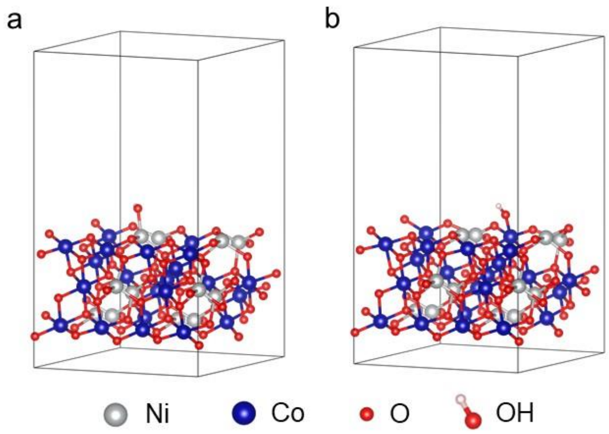

Supplementary Figure 4. The calculation model of OH absorbed on **a**, Ni site and **b**, Co site of $NiCo_2O_4$.

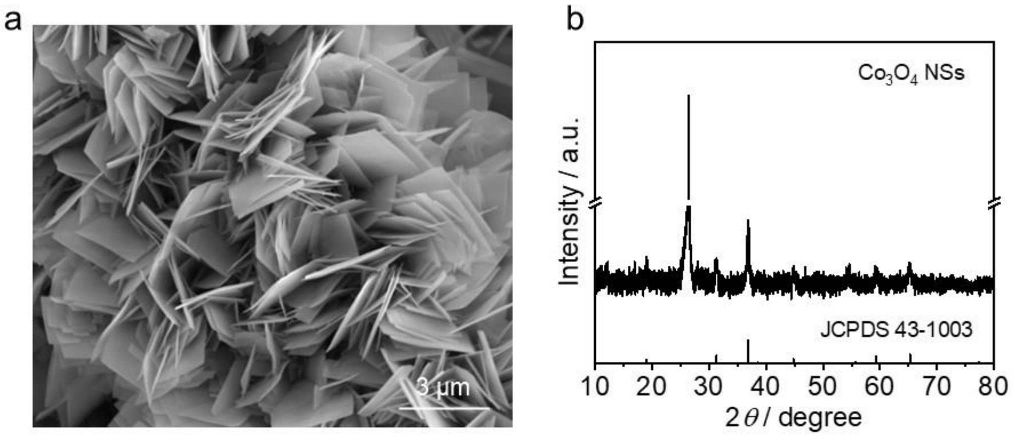

Supplementary Figure 5. a, SEM image and **b**, XRD pattern of Co_3O_4 NSs.

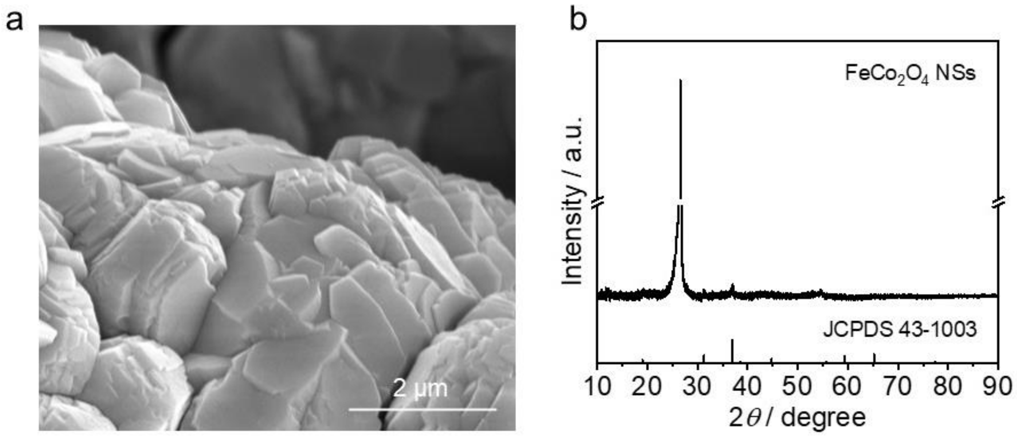

Supplementary Figure 6. a, SEM image and **b**, XRD pattern of $FeCo_2O_4$ NSs.

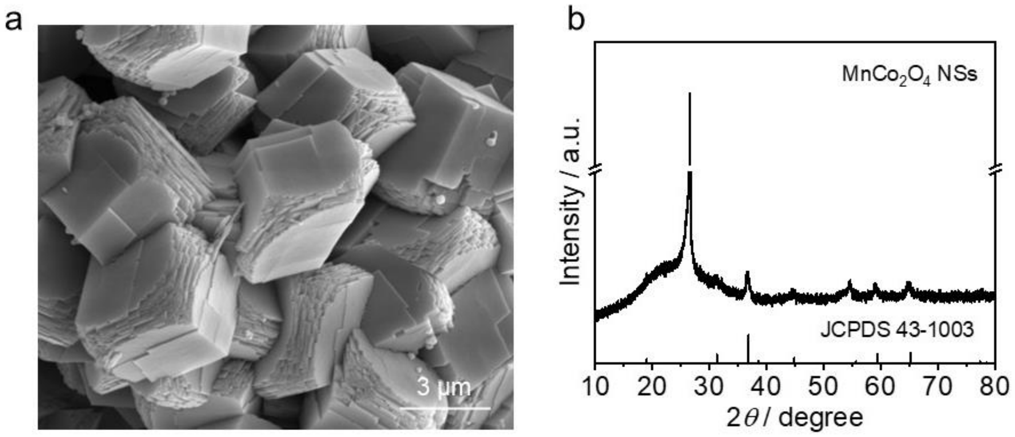

Supplementary Figure 7. a, SEM image and **b**, XRD pattern of $MnCo_2O_4$ NSs.

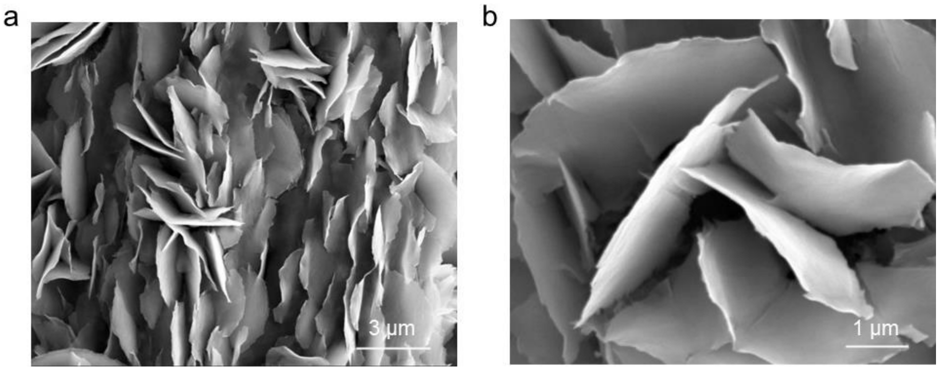

Supplementary Figure 8. a, Low-resolution and **b**, high-resolution SEM images of $NiCo_2O_4$ NSs.

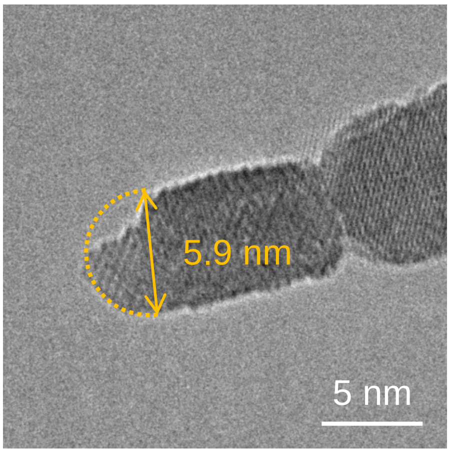

Supplementary Figure 9. High-resolution TEM image of $NiCo_2O_4$ NCs. The curvature radius is ~3.0 nm.

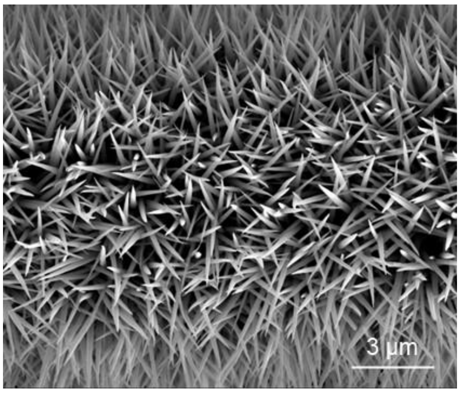

Supplementary Figure 10. Low-resolution SEM image of $NiCo_2O_4$ NCs.

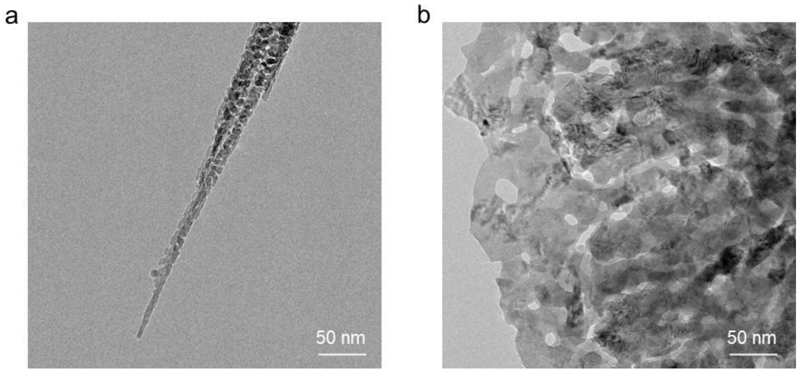

Supplementary Figure 11. a, Low-resolution TEM images of $NiCo_2O_4$ NCs and **b**, $NiCo_2O_4$ NSs.

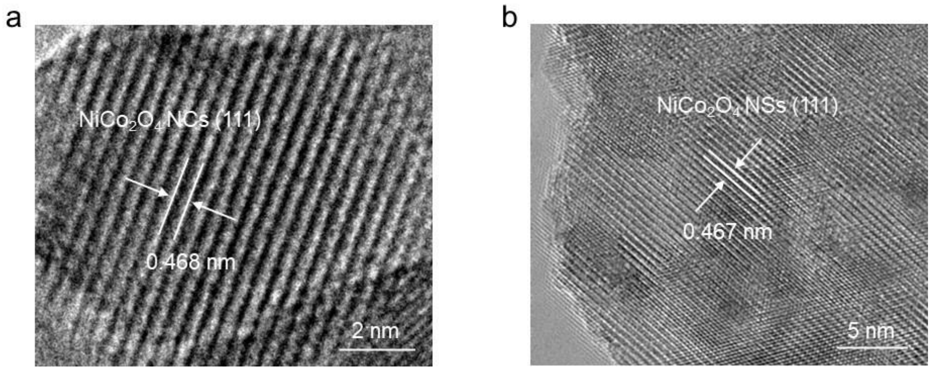

Supplementary Figure 12. a, High-resolution TEM images of $NiCo_2O_4$ NCs and **b**, $NiCo_2O_4$ NSs.

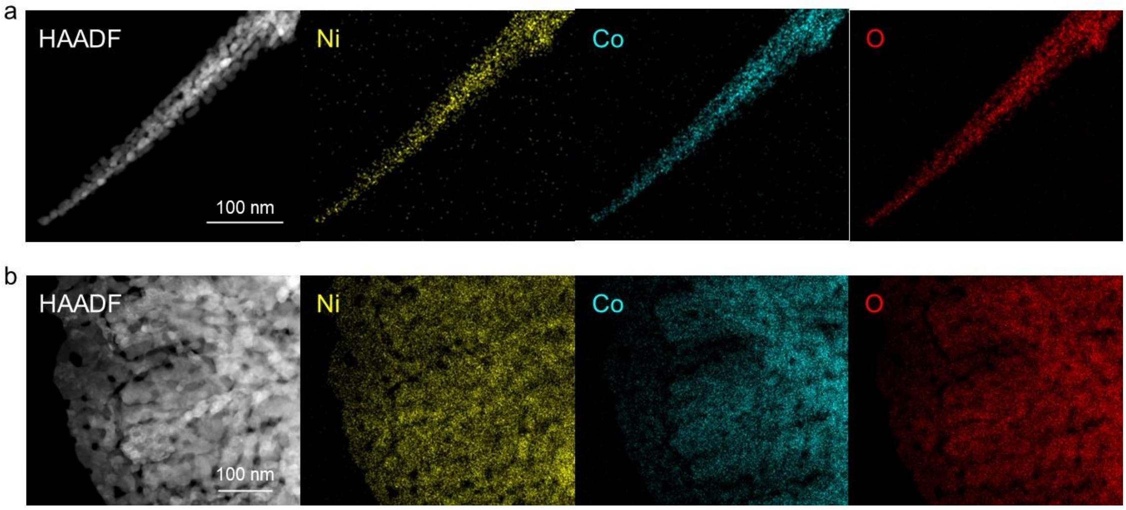

Supplementary Figure 13. a, HAADF and elemental mapping of $NiCo_2O_4$ NCs and **b**, $NiCo_2O_4$ NSs.

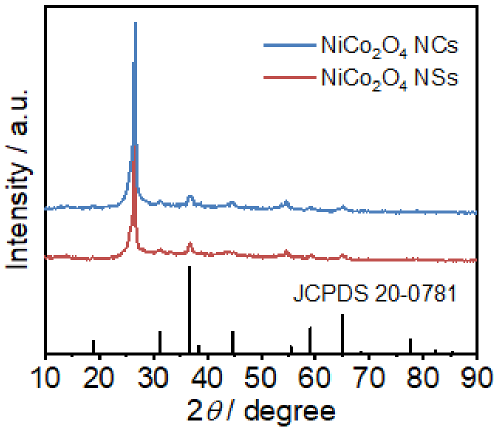

Supplementary Figure 14. XRD pattern of $NiCo_2O_4$ NCs and NSs.

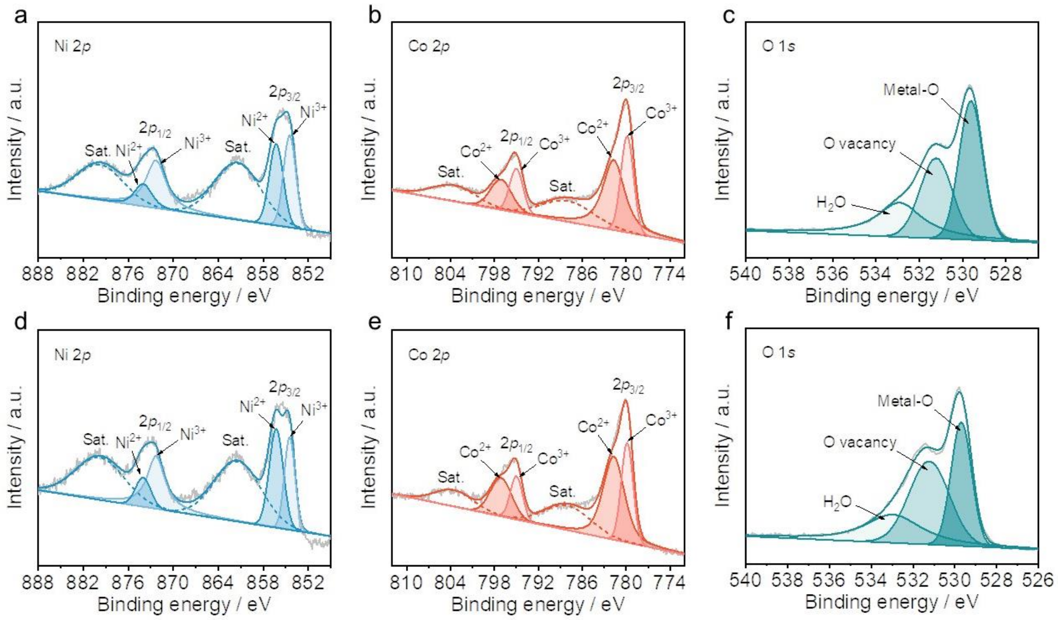

Supplementary Figure 15. **a**, Ni 2*p*, **b**, Co 2*p*, and **c**, O 1*s* XPS spectra of $NiCo_2O_4$ NCs. **d**, Ni 2*p*, **e**, Co 2*p*, and **f**, O 1*s* XPS spectra of $NiCo_2O_4$ NSs.

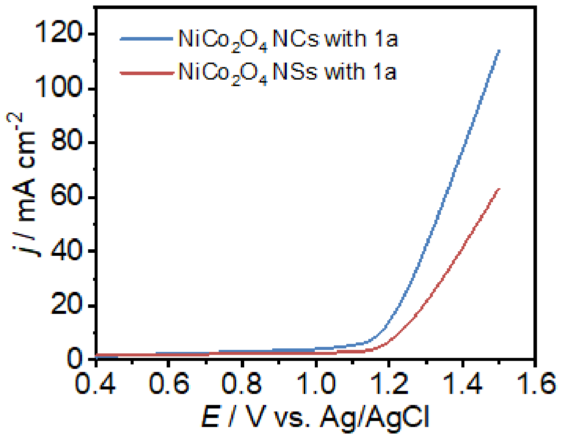

Supplementary Figure 16. LSV curves of **a**, $NiCo_2O_4$ NCs and **b**, $NiCo_2O_4$ NSs.

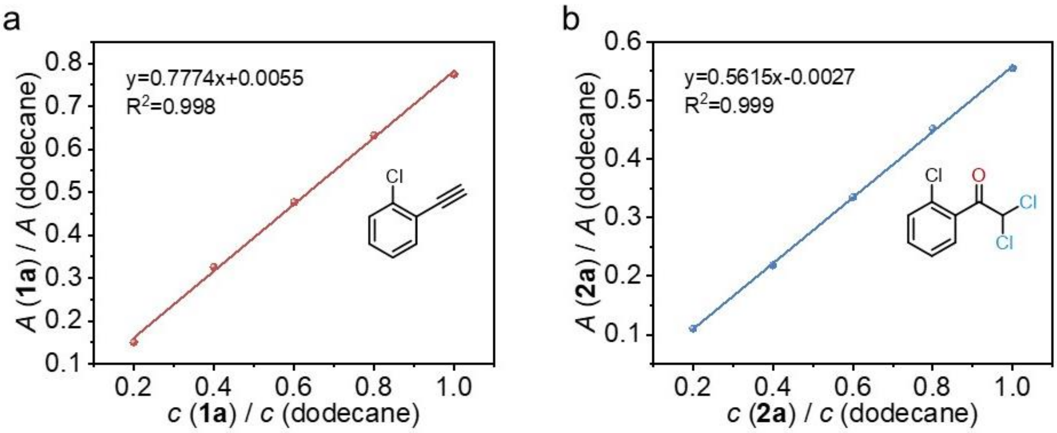

Supplementary Figure 17. The correct calibration curves **1a** and **2a** obtained by adding dodecane as the internal standard for the GC tests.

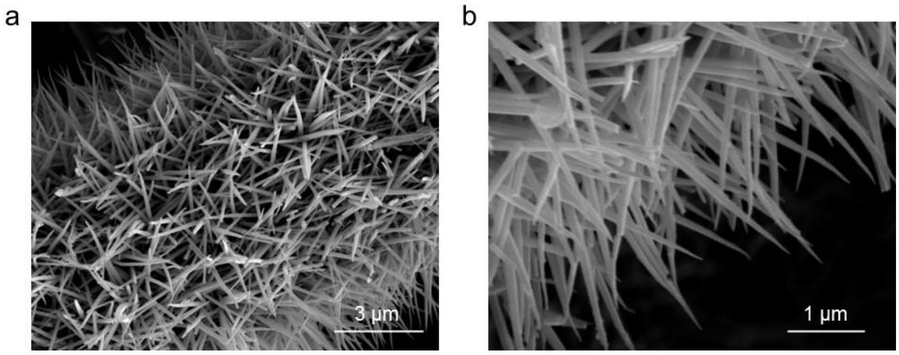

Supplementary Figure 18. **a**, Low-resolution and **b**, high-resolution SEM images of $NiCo_2O_4$ NCs after reaction.

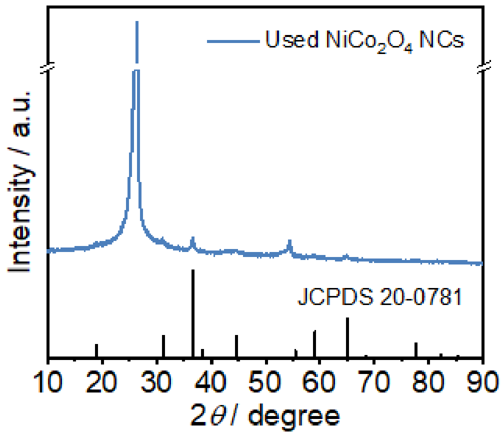

Supplementary Figure 19. XRD pattern of $NiCo_2O_4$ NCs after reaction.

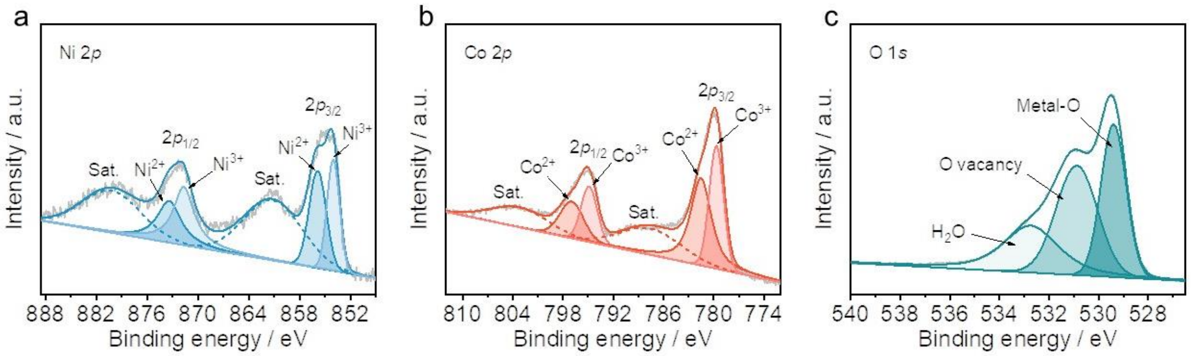

Supplementary Figure 20. a, Ni 2*p*, **b**, Co 2*p*, and **c**, O 1*s* XPS spectra of $NiCo_2O_4$ NCs after reaction.

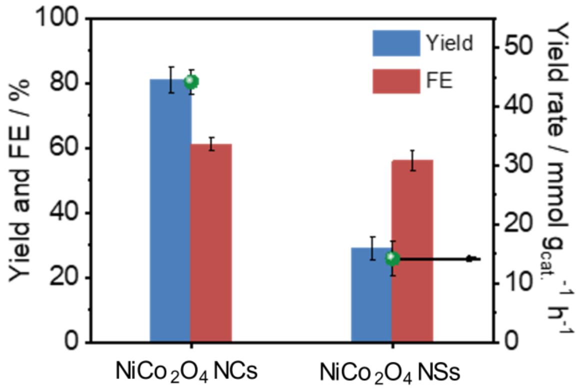

Supplementary Figure 21. The electrosynthesis performance of $NiCo_2O_4$ NCs and NSs at 1.30 V in seawater.

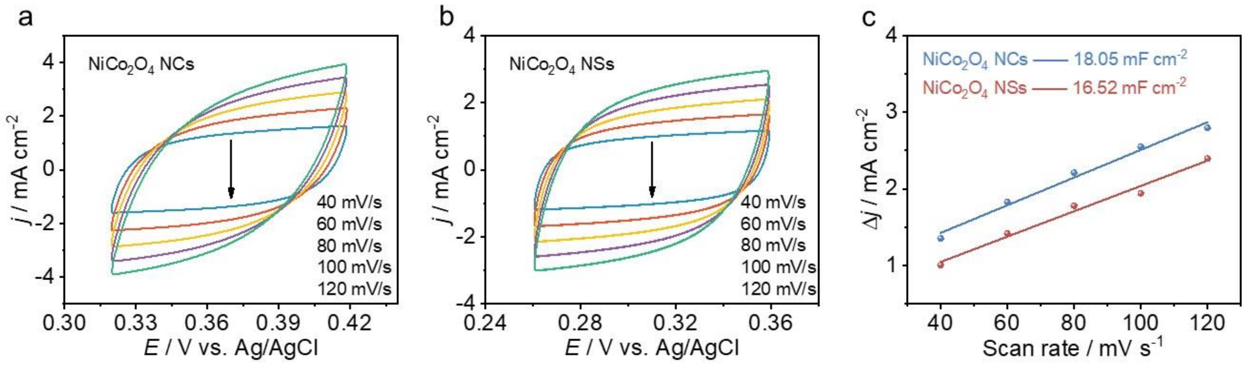

Supplementary Figure 22. CV curves of **a**, $NiCo_2O_4$ NCs and **b**, $NiCo_2O_4$ NSs measured in a non-Faradaic region at the following scan rates: 40, 60, 80, 100, and 120 mV s^{-1}. **c**, Linear fitting of the capacitive currents of the catalysts vs. scan rates.

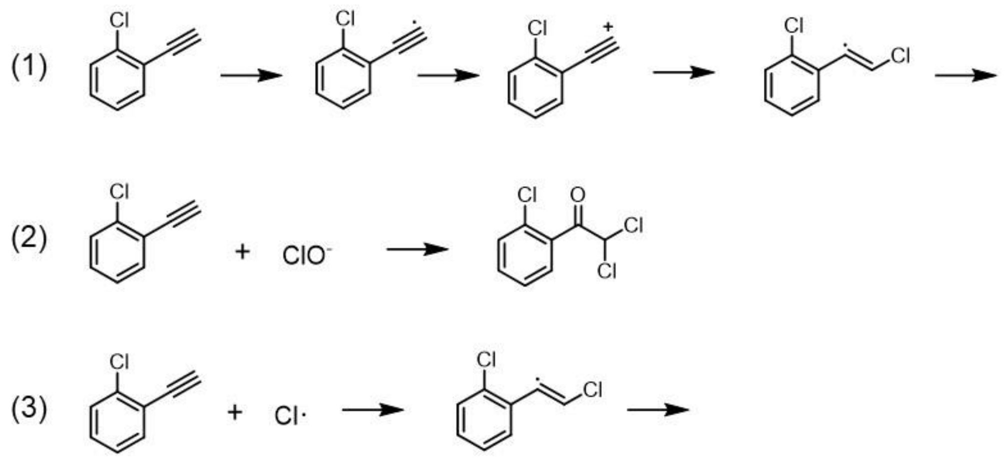

Supplementary Figure 23. The proposed three possible pathways to trigger the oxydichlogenation of alkynes.

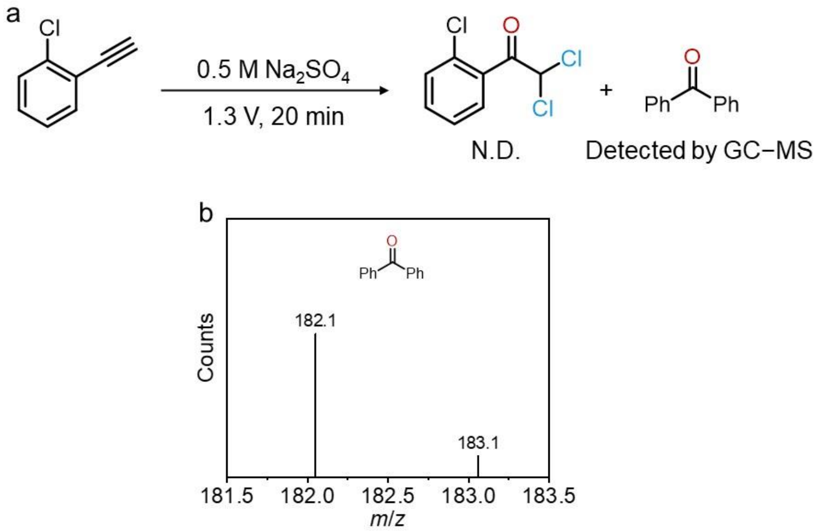

Supplementary Figure 24. a, Control experiment using 0.5 M Na_2SO_4 as the electrolyte solution with 1,1-diphenylethylene addition. **b**, GC−MS analysis of hydroxyl radicals captured by 1,1-diphenylethylene.

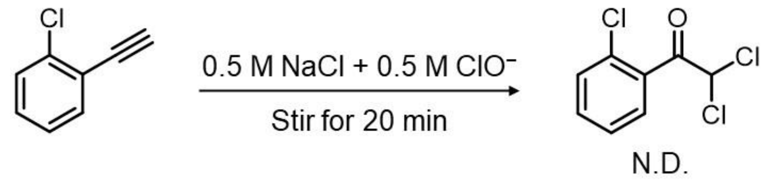

Supplementary Figure 25. Control experiment using 0.5 M NaCl containing 0.5 M ClO^{-} as the electrolyte solution under stirring for 20 min.

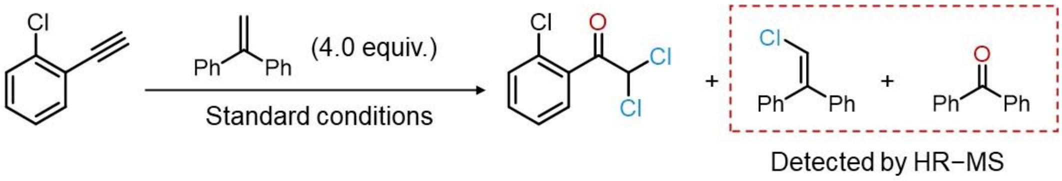

Supplementary Figure 26. Control experiment using 0.5 M NaCl as the electrolyte solution with 1,1-diphenylethylene addition.

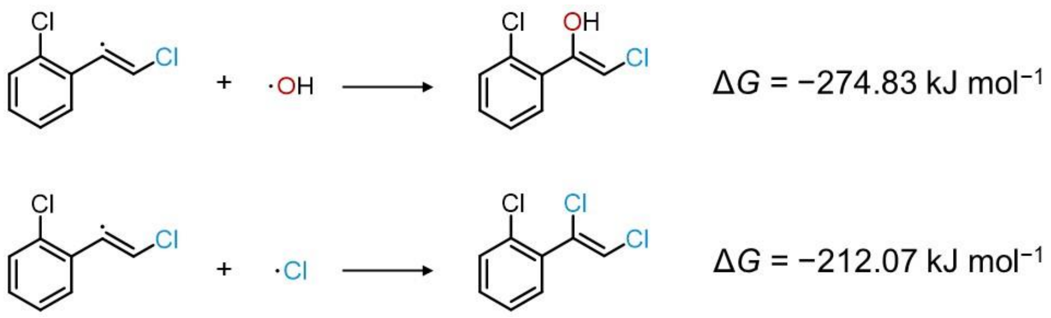

Supplementary Figure 27. The reaction energy of hydroxyl and chloride substitution on vinyl radical.

Supplementary Note 1. Gaussian16 software was used to apply the DFT quantum chemistry calculation method for the competition between hydroxyl substitution and chloride substitution.

B3LYP/6-31G[14] with DFT-D3 correction[15] was used to optimize the geometric structure, and B3LYP/6-311G+[14] was used to obtain the single point energy of the molecule. Compared with the Gibbs free energy of these two reactions, we found that the reaction energy of hydroxyl substitution is 62.76 kJ mol^{-1} greater than that of chloride substitution. In addition, both hydroxyl substitution and chloride substitution are downhill processes. However, hydroxyl radical formation is difficult. Based on the CHE model[16], the formation energy of hydroxyl radicals can be expressed as $G = 2.22 - eU - 0.059$ pH (eV). Fortunately, at the oxidational potential, this reaction would be promoted. For chlorine radicals, there is no potential or pH-dependent effect. All of the above analyses indicate that hydroxyl substitution is easier than chloride substitution.

^{1}H NMR, ^{13}C NMR spectra and GC–MS or HR–MS data

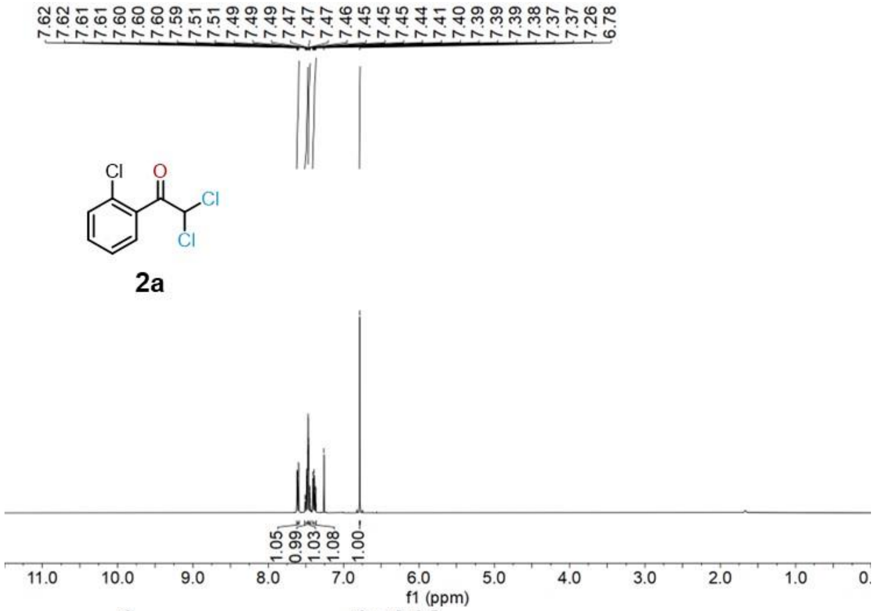

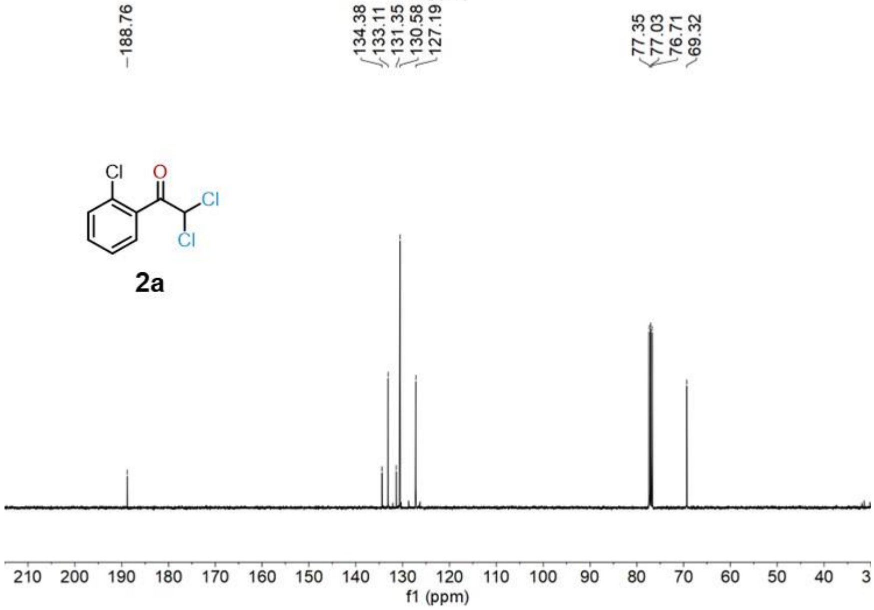

^{1}H NMR (400 MHz, $CDCl_3$) δ [ppm] 7.61 (ddd, J = 7.7, 1.7, 0.7 Hz, 1H), 7.52 – 7.47 (m, 1H), 7.47 – 7.44 (m, 1H), 7.39 (ddd, J = 7.7, 6.7, 2.0 Hz, 1H), 6.78 (s, 1H); **^{13}C NMR** (101 MHz, $CDCl_3$) δ [ppm] 188.76, 134.38, 133.11, 131.35, 130.58, 127.19, 69.32; **GC−MS** (EI) m/z 222.0, and theoretical value for $C_8H_5Cl_3O$ is 221.9.

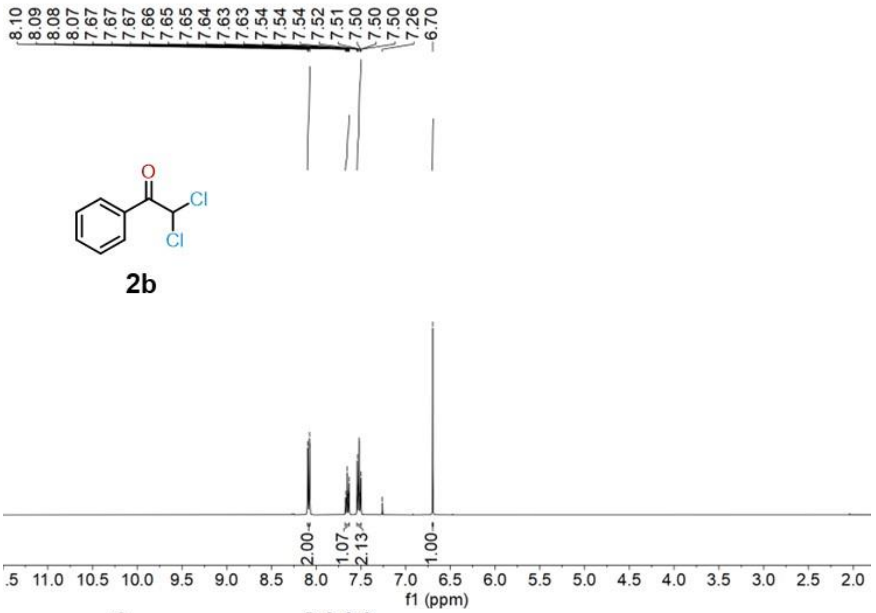

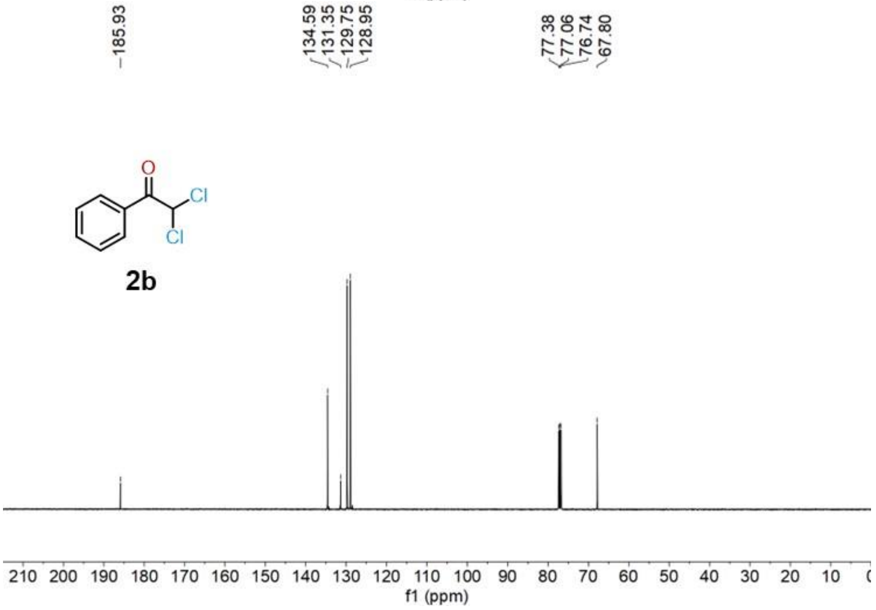

^{1}H NMR (400 MHz, $CDCl_3$) δ [ppm] 8.08 (dd, J = 8.5, 1.3 Hz, 2H), 7.67 – 7.63 (m, 1H), 7.54 – 7.50 (m, 2H), 6.70 (s, 1H); **^{13}C NMR** (101 MHz, $CDCl_3$) δ [ppm] 185.93, 134.59, 131.35, 129.75, 128.95, 67.80; **HR−MS** (ESI−TOF) *m/z* $[M+H]^+$ 188.9901, and theoretical value for $C_8H_6Cl_2O$ is 188.9869.

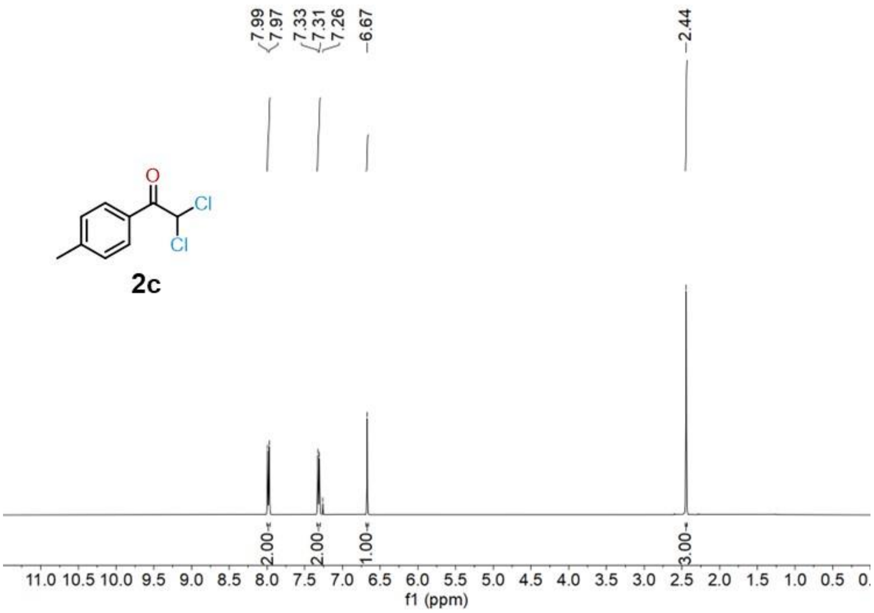

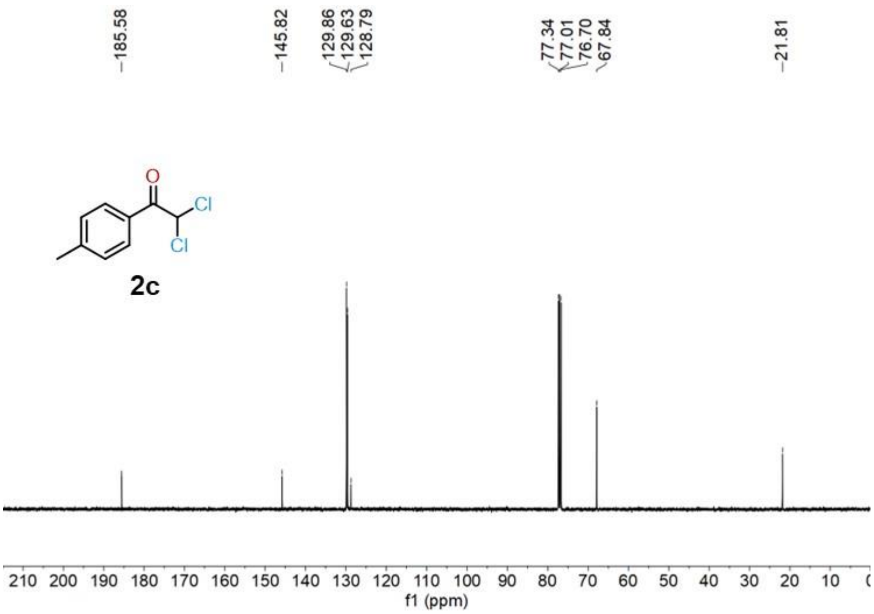

^{1}H NMR (400 MHz, $CDCl_3$) δ [ppm] 7.98 (d, J = 8.4 Hz, 2H), 7.32 (d, J = 8.3 Hz, 2H), 6.67 (s, 1H), 2.44 (s, 3H); **^{13}C NMR** (101 MHz, $CDCl_3$) δ [ppm] 185.58, 145.82, 129.86, 129.63, 128.79, 67.84, 21.81; **GC−MS** (EI) *m/z* 202.0, and theoretical value for $C_9H_8Cl_2O$ is 202.0.

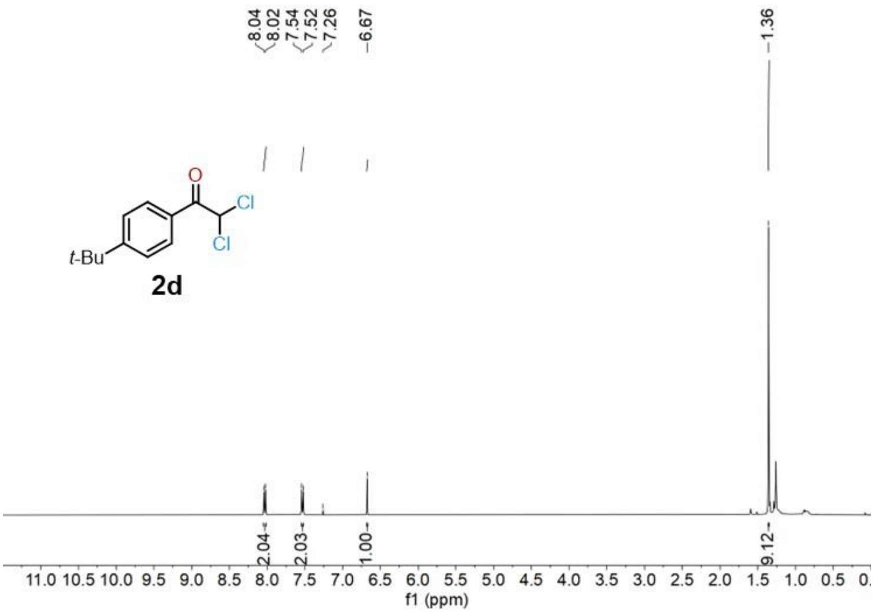

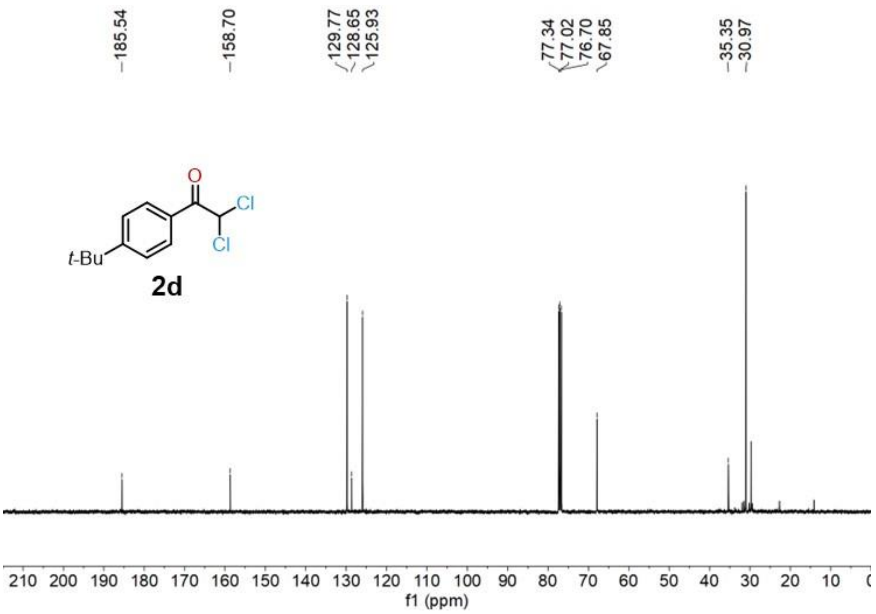

^{1}H NMR (400 MHz, CDCl3) δ [ppm] 8.03 (d, J = 8.8 Hz, 2H), 7.53 (d, J = 8.8 Hz, 2H), 6.67 (s, 1H), 1.36 (s, 9H); **^{13}C NMR** (101 MHz, CDCl3) δ [ppm] 185.54, 158.70, 129.77, 128.65, 125.93, 67.85, 35.35, 30.97; **GC−MS** (EI) m/z 244.0, and theoretical value for $C_{12}H_{14}Cl_2O$ is 244.0.

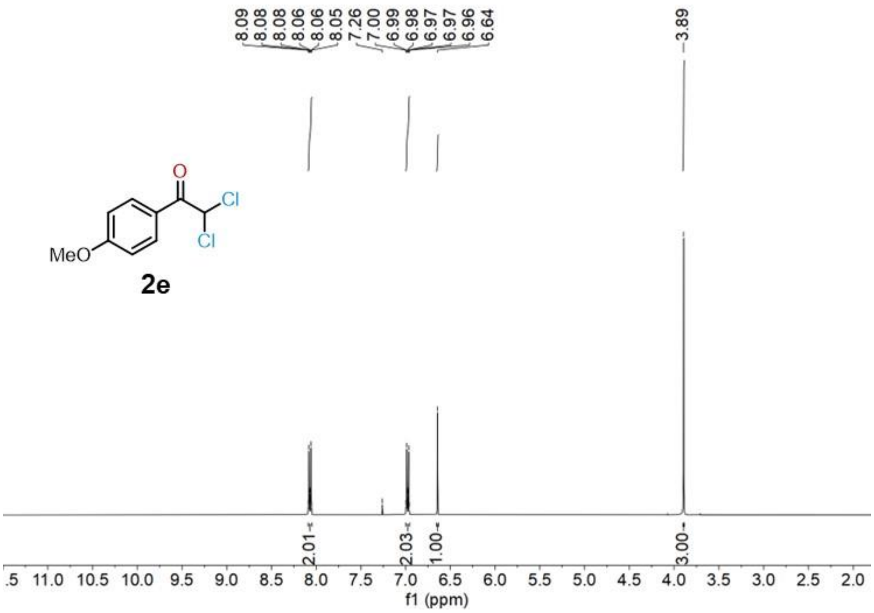

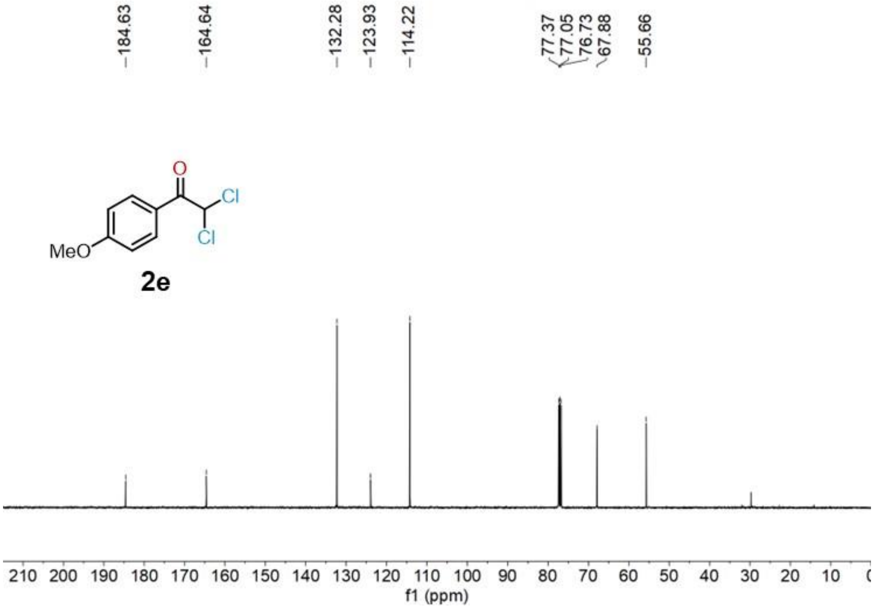

^{1}H NMR (400 MHz, $CDCl_3$) δ [ppm] 8.09 – 8.05 (m, 2H), 7.00 – 6.96 (m, 2H), 6.64 (s, 1H), 3.89 (s, 3H); **^{13}C NMR** (101 MHz, $CDCl_3$) δ [ppm] 184.63, 164.64, 132.28, 123.93, 114.22, 67.88, 55.66; **HR−MS** (ESI−TOF) *m/z* $[M+H]^+$ 236.0243, and theoretical value for $C_9H_8Cl_2O_2$ is 236.0240.

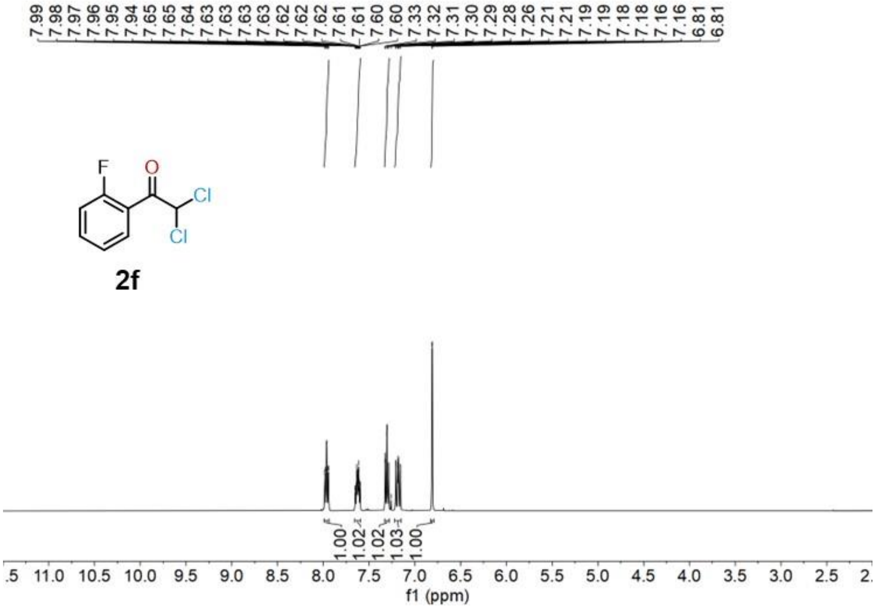

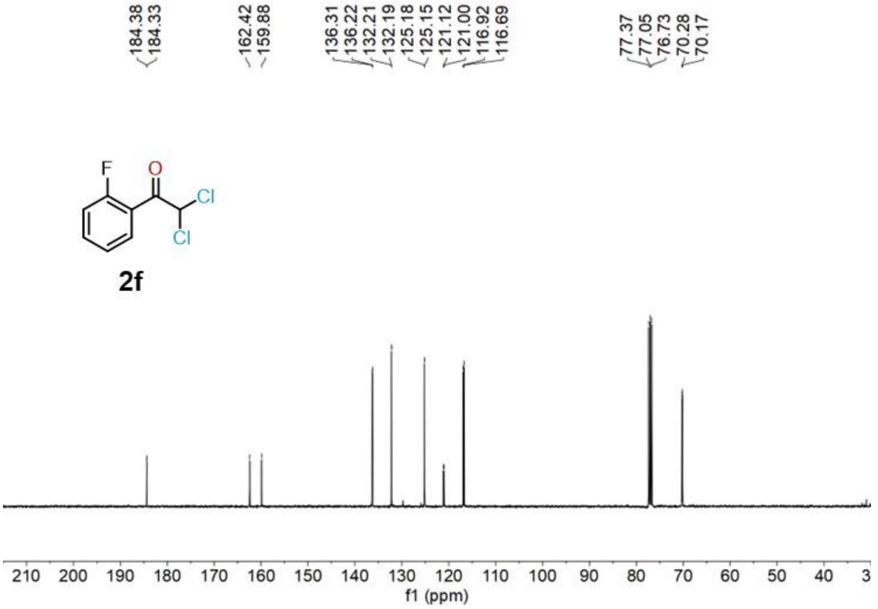

^{1}H NMR (400 MHz, $CDCl_3$) δ [ppm] 7.96 (td, J = 7.6, 1.9 Hz, 1H), 7.62 (dddd, J = 8.3, 7.2, 5.1, 1.9 Hz, 1H), 7.31 (td, J = 7.5, 1.1 Hz, 1H), 6.81 (d, J = 2.0 Hz, 1H); **^{13}C NMR** (101 MHz, $CDCl_3$) δ [ppm] 184.38, 184.34, 162.42, 159.89, 136.32, 136.22, 132.21, 132.19, 125.19, 125.15, 121.13, 121.00, 116.93, 116.70, 70.29, 70.18; **GC−MS** (EI) *m/z* 206.0, and the theoretical value for $C_8H_5Cl_2FO$ is 206.0.

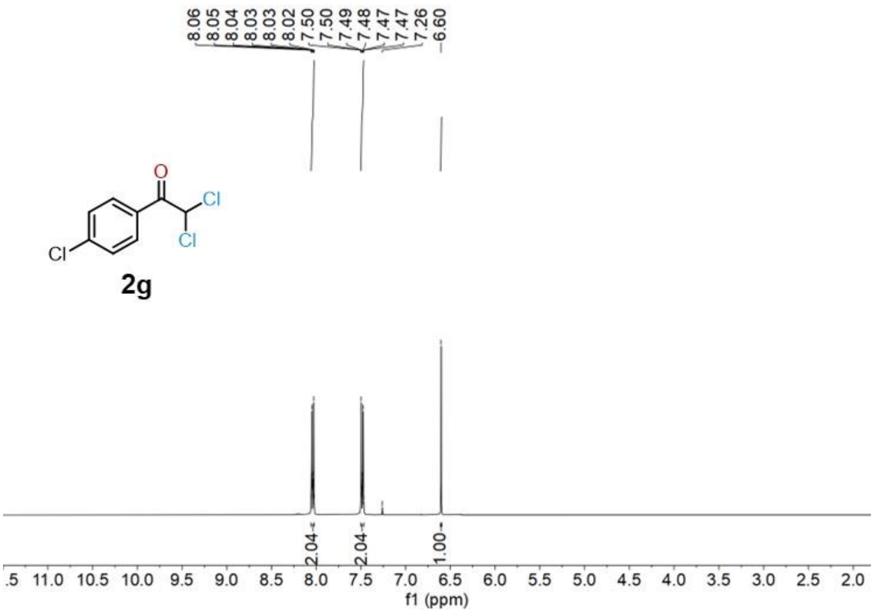

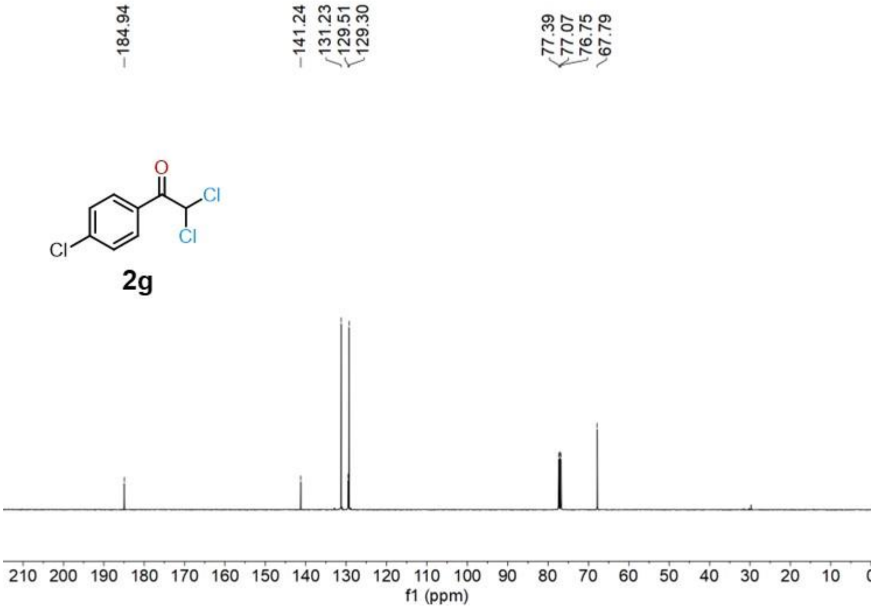

^{1}H NMR (400 MHz, $CDCl_3$) δ [ppm] 8.06 – 8.02 (m, 2H), 7.50 – 7.47 (m, 2H), 6.60 (s, 1H); **^{13}C NMR** (101 MHz, $CDCl_3$) δ [ppm] 184.94, 141.24, 131.23, 129.51, 129.30, 67.79; **GC−MS** (EI) *m/z* 221.9, and theoretical value for $C_8H_5Cl_3O$ is 221.9.

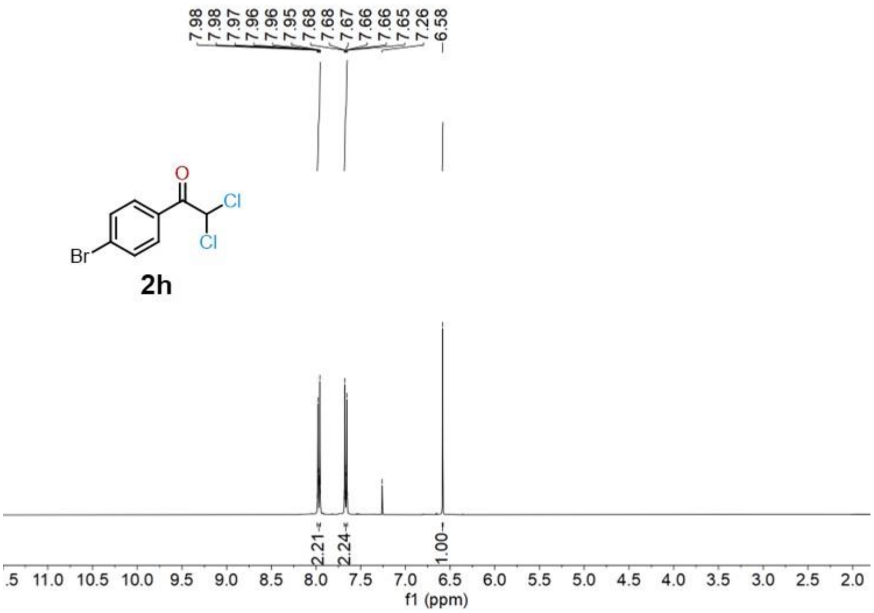

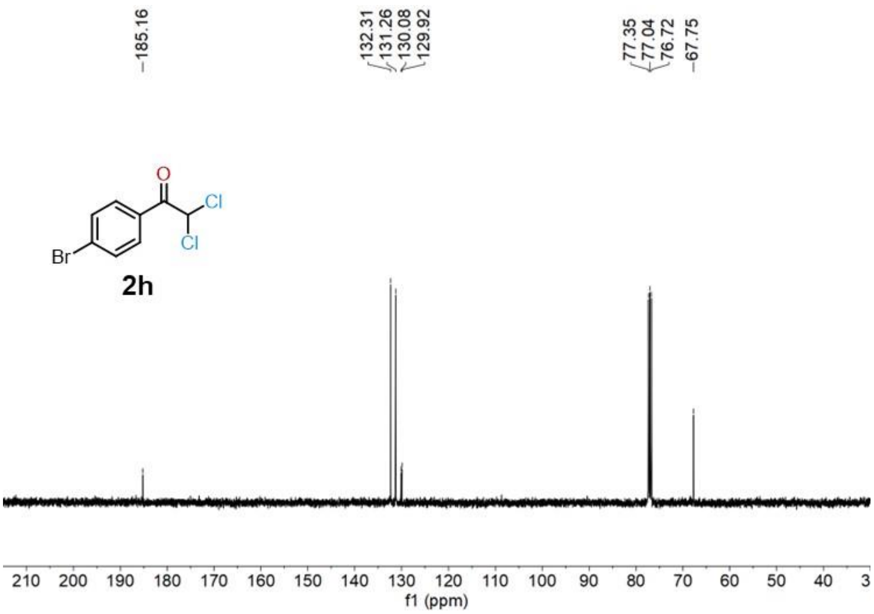

^{1}H NMR (400 MHz, $CDCl_3$) δ [ppm] 7.98 – 7.95 (m, 2H), 7.68 – 7.65 (m, 2H), 6.58 (s, 1H); **^{13}C NMR** (101 MHz, $CDCl_3$) δ [ppm] 185.16, 132.31, 131.26, 130.08, 129.92, 67.75; **GC−MS** (EI) *m/z* 265.9, and theoretical value for $C_8H_5BrCl_2O$ is 265.9.

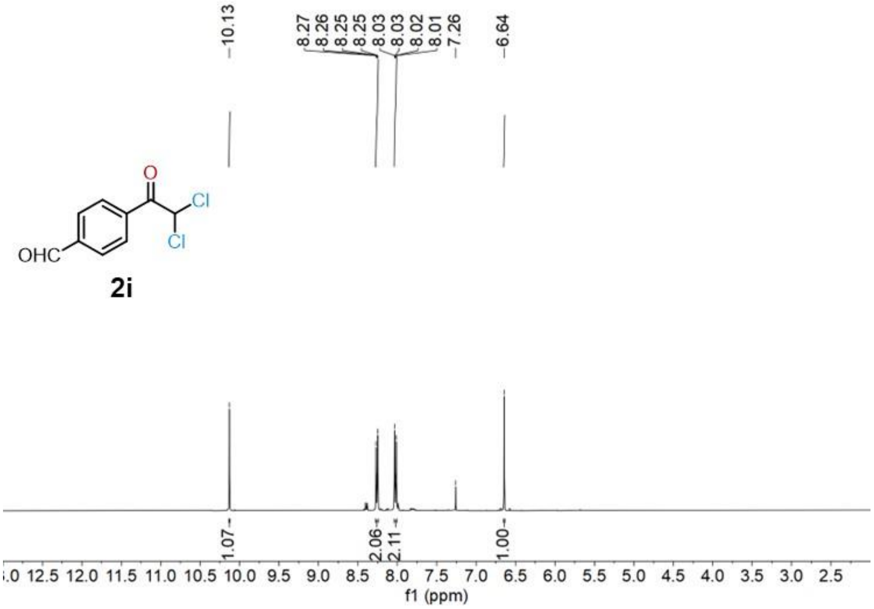

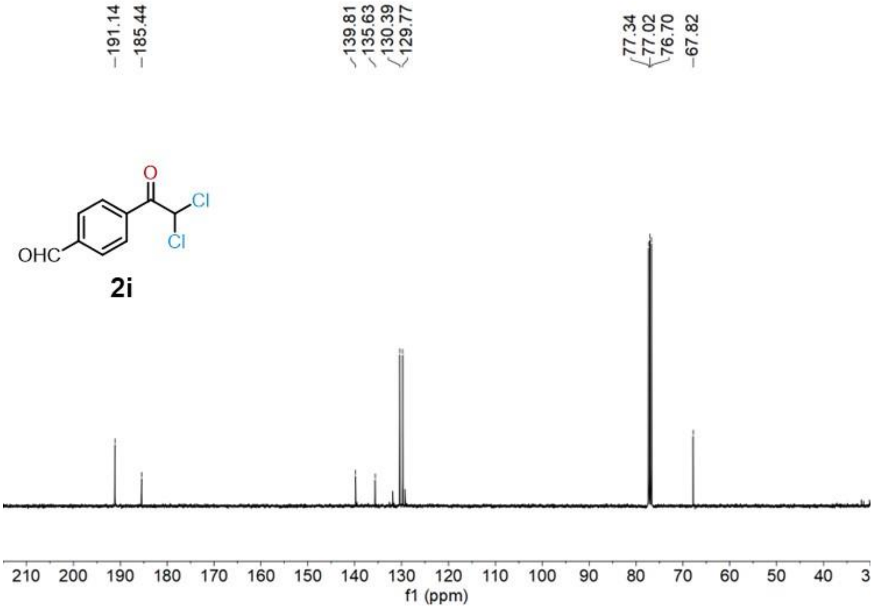

^{1}H NMR (400 MHz, $CDCl_3$) δ [ppm] 10.13 (s, 1H), 8.27 – 8.24 (m, 2H), 8.04 – 8.01 (m, 2H), 6.64 (s, 1H); **^{13}C NMR** (101 MHz, $CDCl_3$) δ [ppm] 191.14, 185.44, 139.81, 135.63, 130.39, 129.77, 67.82; **GC−MS** (EI) *m/z* 216.0, and theoretical value for $C_9H_6Cl_2O_2$ is 216.0.

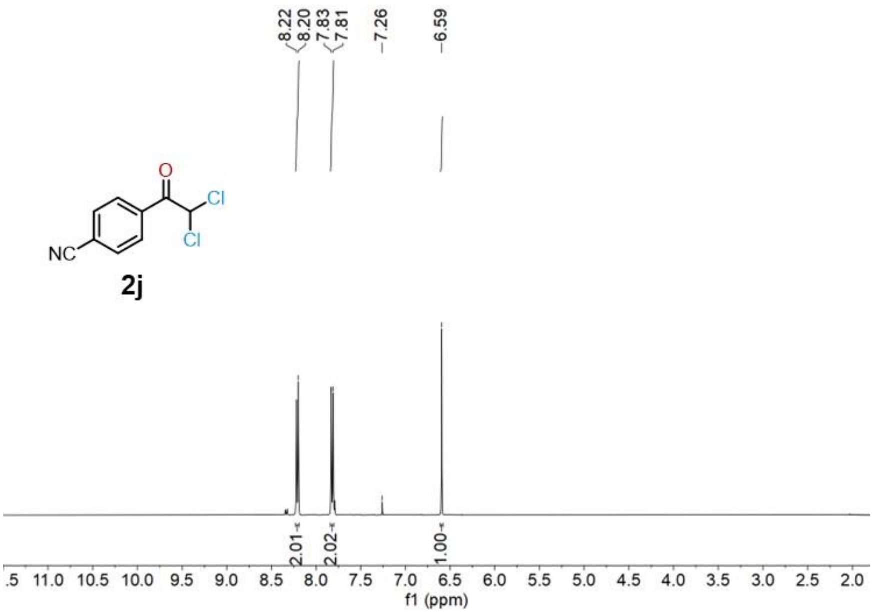

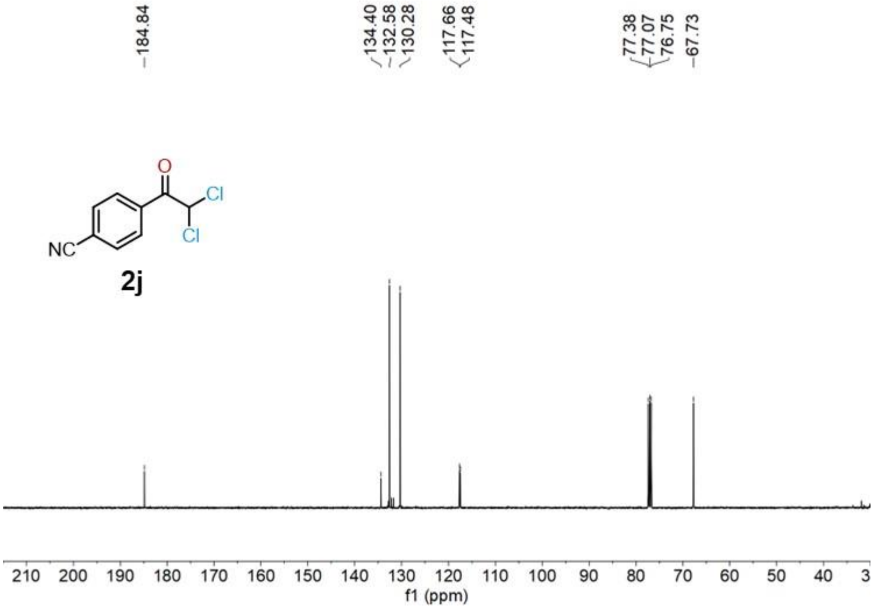

^{1}**H NMR** (400 MHz, $CDCl_3$) δ [ppm] 8.21 (d, J = 8.8 Hz, 2H), 7.82 (d, J = 8.8 Hz, 2H), 6.59 (s, 1H); ^{13}**C NMR** (101 MHz, $CDCl_3$) δ [ppm] 184.84, 134.40, 132.58, 130.28, 117.66, 117.48, 67.73; **GC−MS** (EI) *m/z* 213.0, and theoretical value for $C_9H_5Cl_2NO$ is 213.0.

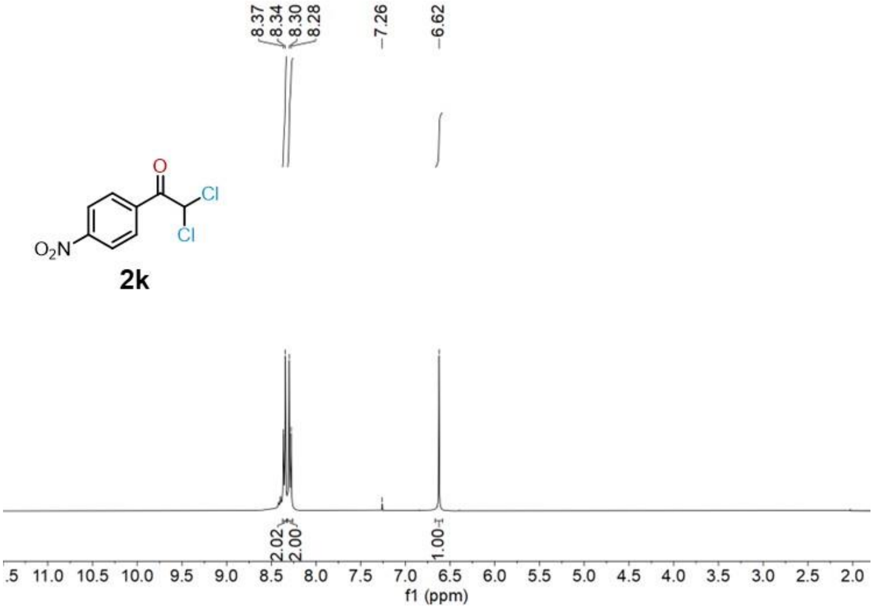

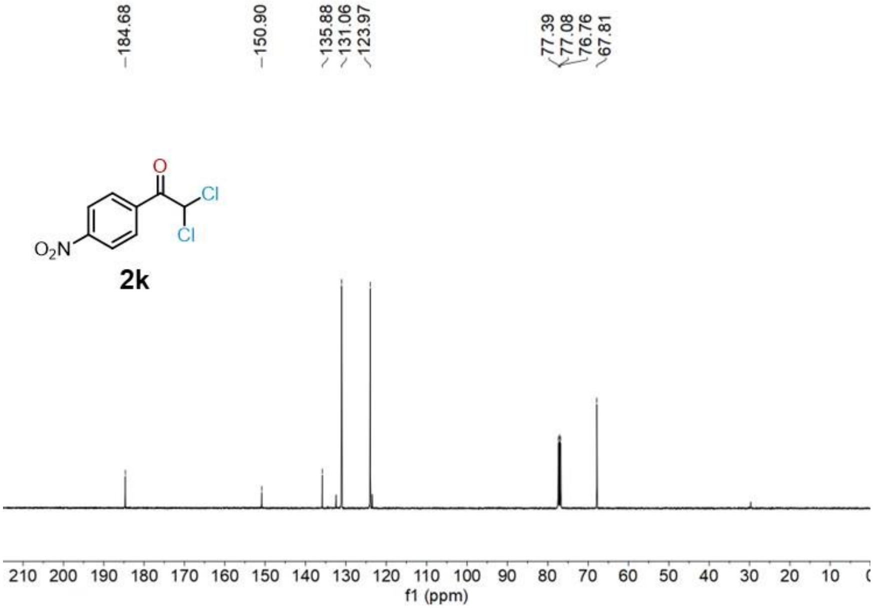

^{1}H NMR (400 MHz, $CDCl_3$) δ [ppm] 8.35 (d, *J* = 9.2 Hz, 2H), 8.29 (d, *J* = 9.0 Hz, 2H), 6.62 (s, 1H); **^{13}C NMR** (101 MHz, $CDCl_3$) δ [ppm] 184.68, 150.90, 135.88, 131.06, 123.97, 67.81; **GC−MS** (EI) *m/z* 233.0, and theoretical value for $C_8H_5Cl_2NO_3$ is 233.0.

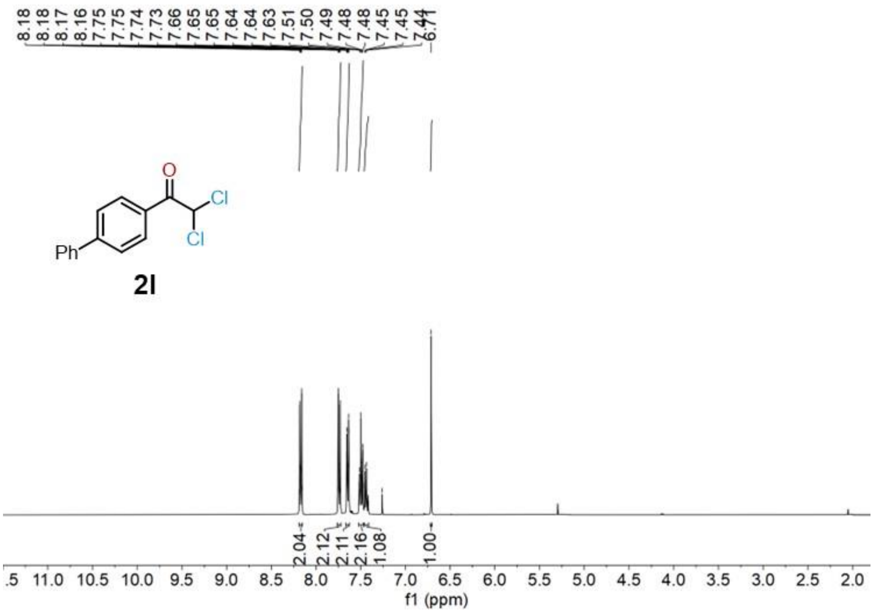

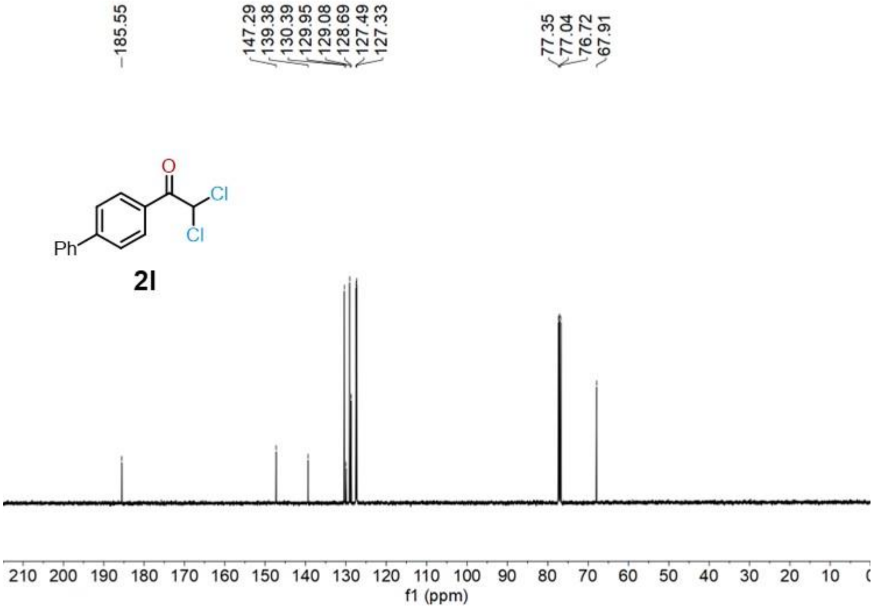

^{1}H NMR (400 MHz, $CDCl_3$) δ [ppm] 8.19 – 8.15 (m, 2H), 7.76 – 7.72 (m, 2H), 7.67 – 7.63 (m, 2H), 7.52 – 7.47 (m, 2H), 7.46 – 7.41 (m, 1H), 6.71 (s, 1H); **^{13}C NMR** (101 MHz, $CDCl_3$) δ [ppm] 185.55, 147.29, 139.38, 130.39, 129.95, 129.08, 128.69, 127.49, 127.33, 67.91; **HR−MS** (ESI−TOF) *m/z* $[M+H]^+$ 265.0178, and theoretical value for $C_{14}H_{10}Cl_2O$ is 265.0182.

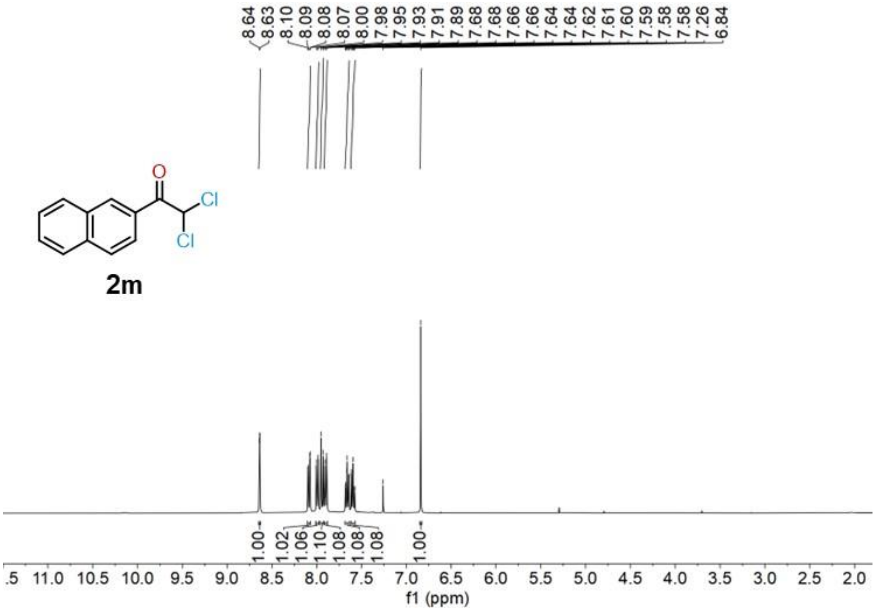

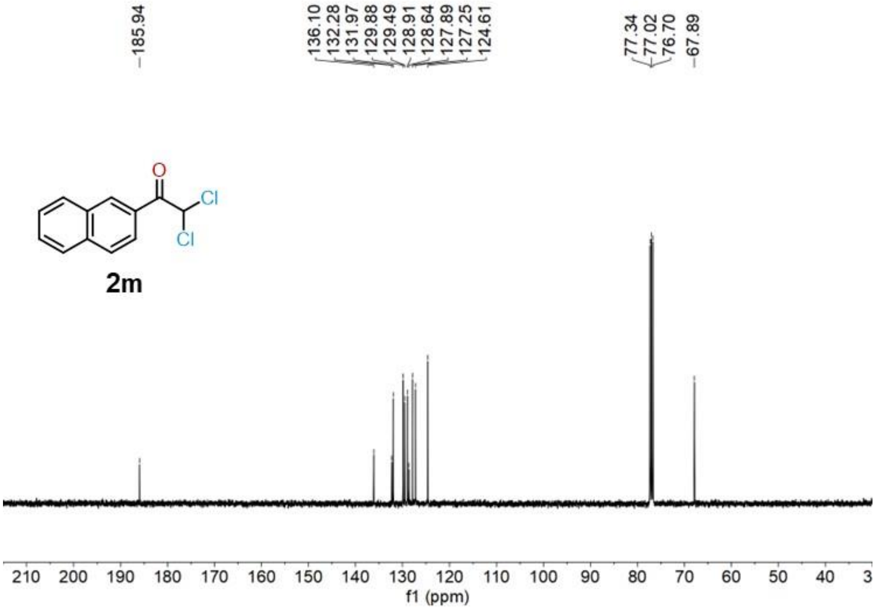

^{1}H NMR (400 MHz, $CDCl_3$) δ [ppm] 8.64 (d, J = 2.3 Hz, 1H), 8.09 (dd, J = 8.7, 1.9 Hz, 1H), 7.99 (d, J = 7.5 Hz, 1H), 7.94 (d, J = 8.7 Hz, 1H), 7.90 (d, J = 8.3 Hz, 1H), 7.66 (td, J = 7.6, 1.4 Hz, 1H), 7.60 (td, J = 7.5, 1.4 Hz, 1H), 6.84 (s, 1H); **^{13}C NMR** (101 MHz, $CDCl_3$) δ [ppm] 185.94, 136.10, 132.28, 131.97, 129.88, 129.49, 128.91, 128.64, 127.89, 127.25, 124.61, 67.89; **GC−MS** (EI) *m/z* 238.0, and theoretical value for $C_{12}H_8Cl_2O$ is 238.0.

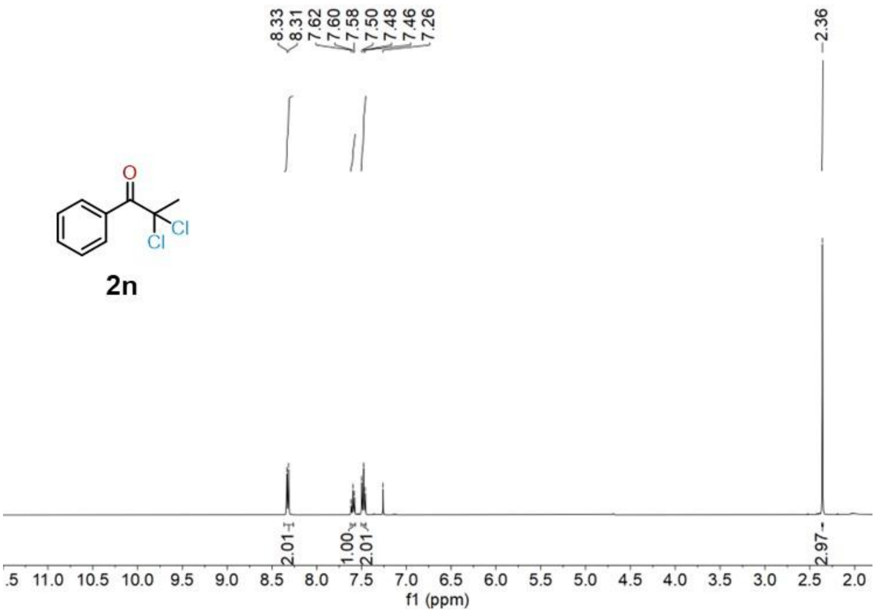

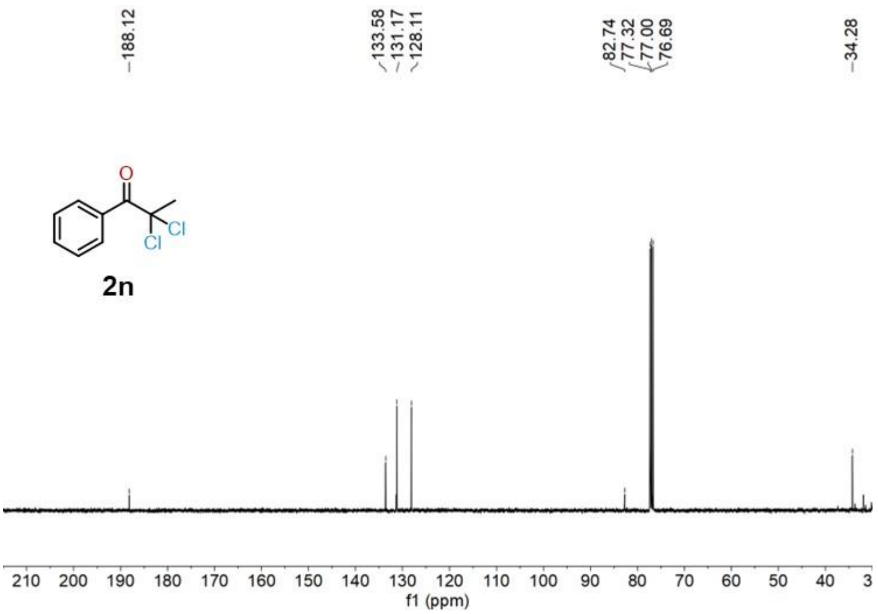

^{1}H NMR (400 MHz, $CDCl_3$) δ [ppm] 8.32 (d, J = 7.3 Hz, 2H), 7.60 (t, J = 7.4 Hz, 1H), 7.48 (t, J = 7.7 Hz, 2H), 2.36 (s, 3H); **^{13}C NMR** (101 MHz, $CDCl_3$) δ [ppm] 188.12, 133.58, 131.17, 128.11, 82.74, 34.28; **HR−MS** (ESI−TOF) *m/z* $[M+H]^+$ 203.0017, and theoretical value for $C_9H_8Cl_2O$ is 203.0025.

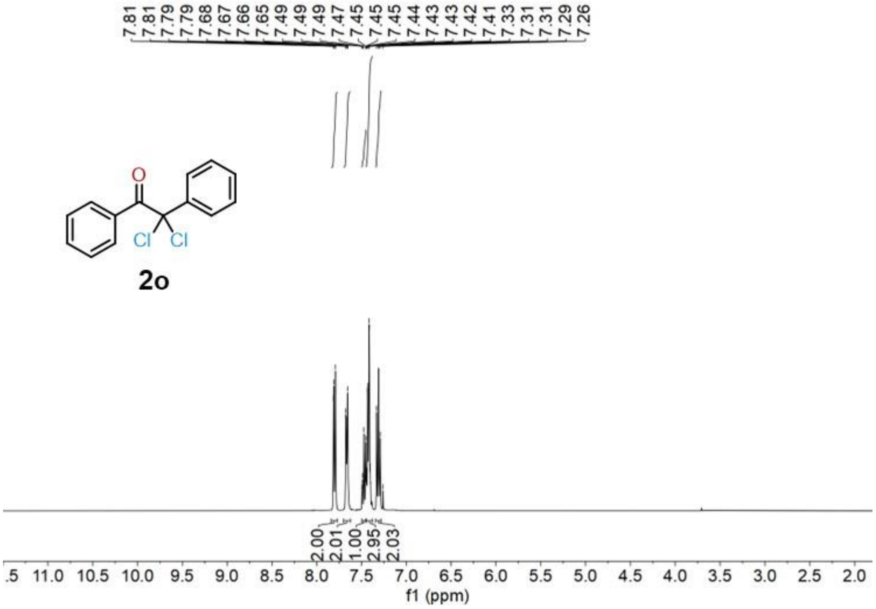

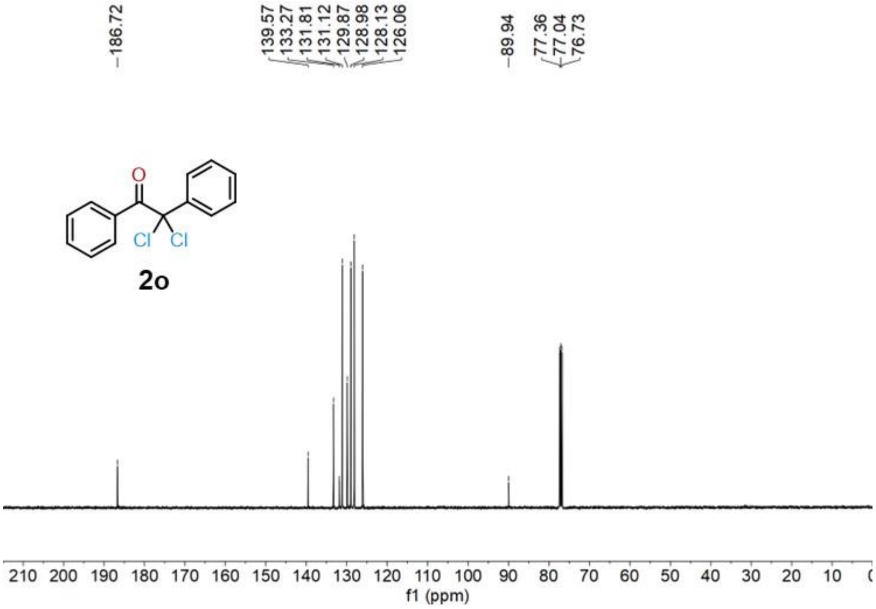

^{1}H NMR (400 MHz, $CDCl_3$) δ [ppm] 7.80 (dd, J = 8.6, 1.3 Hz, 2H), 7.66 (dd, J = 7.8, 1.8 Hz, 2H), 7.50 – 7.45 (m, 1H), 7.42 (dd, J = 7.2, 0.9 Hz, 3H), 7.34 – 7.28 (m, 2H); **^{13}C NMR** (101 MHz, $CDCl_3$) δ [ppm] 186.72, 139.57, 133.27, 131.81, 131.12, 129.87, 128.98, 128.13, 126.06, 89.94; **GC−MS** (EI) *m/z* 264.0, and theoretical value for $C_{14}H_{10}Cl_2O$ is 264.0.

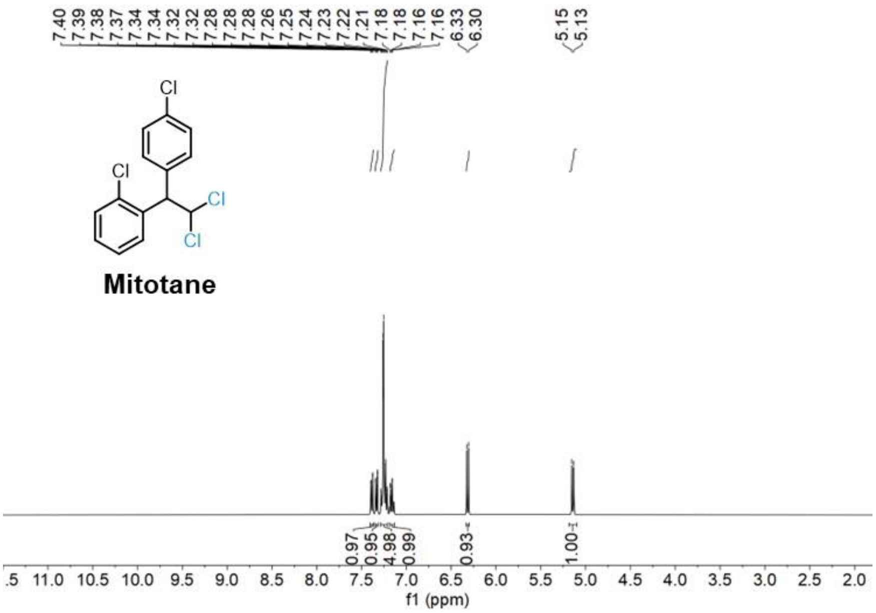

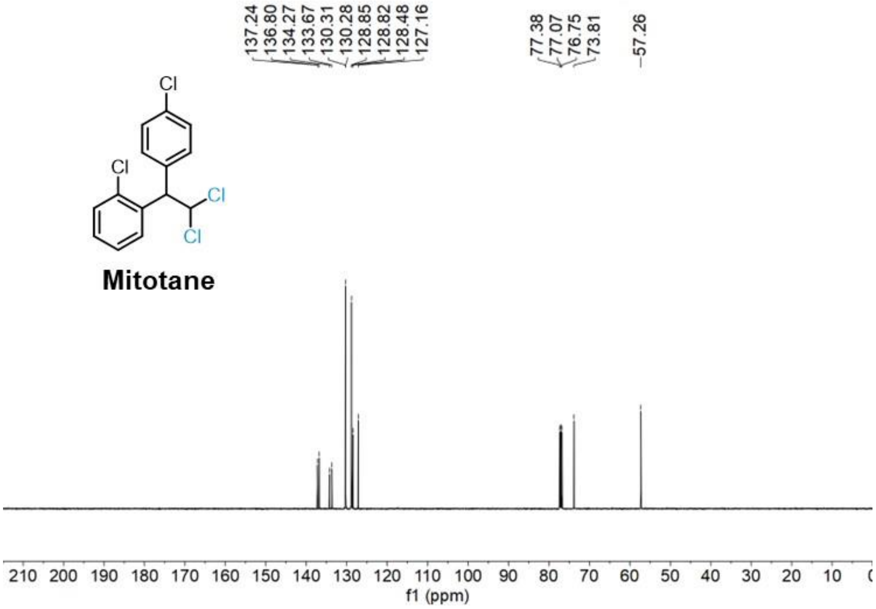

^{1}**H NMR** (400 MHz, $CDCl_3$) δ [ppm] 7.39 (dd, J = 7.8, 1.8 Hz, 1H), 7.33 (dd, J = 7.9, 1.5 Hz, 1H), 7.29 – 7.21 (m, 5H), 7.17 (dd, J = 7.7, 1.8 Hz, 1H), 6.32 (d, J = 8.7 Hz, 1H), 5.14 (d, J = 8.7 Hz, 1H); ^{13}**C NMR** (101 MHz, $CDCl_3$) δ [ppm] 137.24, 136.80, 134.27, 133.67, 130.31, 130.28, 128.85, 128.82, 128.48, 127.16, 73.81, 57.26; **HR−MS** (ESI−TOF) m/z $[M+Na]^+$ 342.9369, and the theoretical value for $C_{14}H_{10}Cl_4$ is 342.9400.

Supplementary References

(1) Zhang, H.; Lu, C.; Hou, H.; Ma, Y.; Yuan, S. Facile Morphology-Controlled Synthesis of Co_3O_4 Nanostructure on Carbon Cloth and Their Morphology-Dependent Pseudocapacitive Performances. *J. Alloys Compd.* **2019**, 797, 970−977.

(2) Adhikari, S.; Noh, G. H.; Kim, D. H. Core-Shell 2D/2D $FeCo_2O_4@Ni(OH)_2$ Nano-on-Microsheet Array Architecture for Excellent Asymmetric Supercapacitor Performance. *Appl. Surf. Sci.* **2023**, 611, 155449.

(3) Li, Y.; Mou, T.; Lu, L.; Jiang, X. Visible-Light-Promoted Oxidative Halogenation of Alkynes. *Chem. Commun.* **2019**, 55, 14299.

(4) Gao, Y.; Yang, R.; Wang, C.; Liu, C.; Wu, Y.; Li, H.; Zhang, B. Field-Induced Reagent Concentration and Sulfur Adsorption Enable Efficient Electrocatalytic Semihydrogenation of Alkynes. *Sci. Adv.* **2022**, 8, abm9477.

(5) Liu, M.; Pang, Y.; Zhang, B.; Luna, P. D.; Voznyy, O.; Xu, J.; Zheng, X.; Dinh, C. T.; Fan, F.; Cao, C. et al. Enhanced Electrocatalytic CO_2 Reduction via Field-Induced Reagent Concentration. *Nature* **2016**, 537, 382−386.

(6) Yang, B.; Liu, K.; Li, H.; Liu, C.; Fu, J.; Li, H.; Huang, J.; Ou, P.; Alkayyali, T.; Cai, C. et al. Accelerating CO_2 Electroreduction to Multicarbon Products via Synergistic Electric–Thermal Field on Copper Nanoneedles. *J. Am. Chem. Soc.* **2022**, 144, 3039−3049.

(7) Xiong, G.; He, P.; Lyu, Z.; Chen, T.; Huang, B.; Chen, L.; Fisher, T. Bioinspired Leaves-on-Branchlet Hybrid Carbon Nanostructure for Supercapacitors. *Nat. Commun.* **2018**, 9, 790.

(8) Huang, Y.; Lu, B.; Xu, J.; Zhang, G.; Qin, L.; Chen, S.; Kong, F. Novel Cross-Linking Poly(Ethylene Oxide) Grafted Poly(1-Hydroxy-2-Methoxyphenol) Copolymers by Secondary Polymerization. *Electrochim. Acta* **2012**, 77, 163−170.

(9) Kresse, G.; Furthmüller, J. Efficient Iterative Schemes for Ab Initio Total-Energy Calculations Using a Plane-Wave Basis Set. *Phys. Rev. B* **1996**, 54, 11169.

(10) Blöchl, P. E. Projector Augmented-Wave Method. *Phys. Rev. B* **1994**, 50, 17953.

(11) Perdew, J. P.; Burke, K.; Ernzerhof, M. Generalized Gradient Approximation Made Simple. *Phys. Rev. Lett.* **1996**, 77, 3865.

(12) Gao, L.; Cui, X.; Wang, Z.; Lin, Z. Operando Unraveling Photothermal-Promoted Dynamic Active-Sites Generation in $NiFe_2O_4$ for Markedly Enhanced Oxygen Evolution. *Proc. Natl. Acad. Sci. U.S.A.* **2021**, 118, e2023421118.

(13) Fishman, M.; Zhuang, H.; Mathew, K.; Dirschka W.; Hennig, R. G. Accuracy of Exchange-Correlation Functionals and Effect of Solvation on the Surface Energy of Copper. *Phys. Rev. B* **2013**, 87, 245402.

(14) Hill, J. G. Gaussian Basis Sets for Molecular Applications. *Int J Quantum Chem* **2013**, 113, 21−34.

(15) Goerigk, L.; Hansen, A.; Bauer, C.; Ehrlich, S.; Najibi, A.; Grimme, S. A Look at the Density Functional Theory Zoo with the Advanced GMTKN55 Database for General Main Group Thermochemistry, Kinetics and Noncovalent Interactions. *Phys. Chem. Chem. Phys* **2017**, 19, 32184−32215.

(16) Nørskov, J. K. Origin of the Overpotential for Oxygen Reduction at a Fuel-Cell Cathode. *J. Phys. Chem. B* **2004**, 108, 17886−17892.
